# The blood-brain barrier: an engineering perspective

**DOI:** 10.3389/fneng.2013.00007

**Published:** 2013-08-30

**Authors:** Andrew D. Wong, Mao Ye, Amanda F. Levy, Jeffrey D. Rothstein, Dwight E. Bergles, Peter C. Searson

**Affiliations:** ^1^Department of Materials Science and Engineering, Johns Hopkins UniversityBaltimore, MD, USA; ^2^Institute for Nanobiotechnology, Johns Hopkins UniversityBaltimore, MD, USA; ^3^The Solomon H. Snyder Department of Neuroscience, Johns Hopkins UniversityBaltimore, MD, USA; ^4^Brain Sciences Institute, Johns Hopkins UniversityBaltimore, MD, USA

**Keywords:** blood-brain barrier, neurovascular unit, capillary, microvasculature, transport

## Abstract

It has been more than 100 years since Paul Ehrlich reported that various water-soluble dyes injected into the circulation did not enter the brain. Since Ehrlich's first experiments, only a small number of molecules, such as alcohol and caffeine have been found to cross the blood-brain barrier, and this selective permeability remains the major roadblock to treatment of many central nervous system diseases. At the same time, many central nervous system diseases are associated with disruption of the blood-brain barrier that can lead to changes in permeability, modulation of immune cell transport, and trafficking of pathogens into the brain. Therefore, advances in our understanding of the structure and function of the blood-brain barrier are key to developing effective treatments for a wide range of central nervous system diseases. Over the past 10 years it has become recognized that the blood-brain barrier is a complex, dynamic system that involves biomechanical and biochemical signaling between the vascular system and the brain. Here we reconstruct the structure, function, and transport properties of the blood-brain barrier from an engineering perspective. New insight into the physics of the blood-brain barrier could ultimately lead to clinical advances in the treatment of central nervous system diseases.

## The physics of the blood-brain barrier (BBB)

### Powering the brain

The BBB is the interface between the vascular system and the brain, and hence we begin by reviewing the architecture of the brain vasculature. The human brain is comprised of ~100 billion neurons and consumes about 15–20 W power. The metabolic nutrients that supply the power are oxygen and glucose. The brain, along with the liver and GI tract, are the most energy expensive organs in the human body. Overall the brain accounts for 15–20% of the base metabolic rate (BMR), consuming 15–20% of oxygen leaving the heart, and 15–20% of the glucose consumed daily (Aiello and Wheeler, 1995; Attwell and Laughlin, 2001; Fish and Lockwood, 2003; Lennie, 2003; Navarrete et al., 2011). Since the brain does not have significant capacity to store metabolic nutrients, fuel to power the brain is provided on-demand by the lungs, and GI system which transfer oxygen and glucose, respectively, to the vascular system. Therefore, the role of the vascular system is crucial in delivering nutrients necessary to maintain normal brain function. Interruption of cerebral blood flow very quickly results in neuronal death; after cardiac arrest apoptosis of neurons begins almost immediately, and brain damage occurs after about 5 min (Hossmann, 2006).

Most of the energy consumed by ATP hydrolysis in the brain is used by neurons for generating nerve impulses (e.g., voltage- and ligand-gated ion channels) and for maintaining ion gradients (e.g., sodium/potassium pumps; Attwell and Laughlin, 2001; Shulman et al., 2004; Raichle and Mintun, 2006). The cerebral metabolic rate (CMR) for ATP utilization in the human brain is about 9.5 μmol g^−1^ s^−1^ in gray matter and about 3 μmol g^−1^ s^−1^ in white matter (Zhu et al., 2012). Therefore, about 77% of the brain's energy consumption is in cortical gray matter, which represents about 50% of the brain volume (Zhu et al., 2012). The gray matter consists of neurons, dendrites, unmyelinated axons, glial cells, and capillaries, whereas white matter is mostly myelinated axons, glial cells, and capillaries. Due to the increased energy demands, the capillary density is 2–4 times higher in gray matter (Borowsky and Collins, 1989b; Heinzer et al., 2008). Gray matter has a higher density of synapses and higher levels of neural activity than white matter and hence increased energy consumption is expected (Zhu et al., 2012). As we show below, the architecture of the brain microvasculature is dictated in large part by the energy needs of the neurons in the brain.

### Brain microvasculature across species

The architecture of the brain microvasculature across species is remarkably similar. The BMR of numerous species (both warm blooded and cold-blooded organisms) follows Keliber's law where the BMR is proportional to body weight with an exponent of 0.75 (Kleiber, 1947). The oxygen (mL min^−1^) and glucose (μmol min^−1^) CMRs increase with brain volume across species, with an exponent of 5/6 (0.85), indicating that the brain is a major energy consumer (Karbowski, 2007, 2009, 2011). The metabolic rate of the human brain, normalized to its mass is about 11 W kg^−1^, almost an order of magnitude larger than that of the human body of 1.3 W kg^−1^ (Aiello and Wheeler, 1995).

From the exponent of 5/6, it follows that the CMR normalized by brain volume scales with brain volume (*V*) with an exponent of −1/6 (CMR/*V* ∝ *V*^−1/6^; Karbowski, 2011). Cerebral blood flow, normalized to brain volume, also has an exponent of −1/6 (CBF/*V* ∝ *V*^−1/6^; Karbowski, 2011), illustrating that cerebral blood flow is directly proportional to CMR across species (CMR ∝ CBF). Indeed, studies in rat brains have shown a strong correlation between local blood flow, glucose utilization, and capillary density (Klein et al., 1986; Borowsky and Collins, 1989a).

The neuron density in the brain scales with brain volume with an exponent of −1/6 (ρ_*n*_ ∝ *V*^−1/6^; Karbowski, 2011). The negative exponent reflects the difficulty in wiring and powering 3D architectures with increasing brain size. The capillary length density also exhibits an exponent of −1/6 (ρ_*c*_ ∝ *V*^−1/6^) showing that the number of neurons is proportional to the total length of capillaries across species (Karbowski, 2011). Equivalently, the capillary length density per neuron is constant across species. The average capillary diameter is only weakly dependent on brain volume with an exponent of 0.08 (*d*_*c*_ ∝ *V*^0.08^), increasing from about 4 μm in the rat brain to about 7 μm in the human brain.

The fact that cerebral blood flow is directly proportional to CMR and that the capillary length density per neuron is constant across species provides evidence that the architecture of the human brain microvasculature is not unique. Neural function is constrained in part by energy demands and hence the spatial distribution of capillaries is closely correlated with metabolic function.

### Microvasculature of the human brain

The average adult human brain weighs about 1500 g and occupies about 1200 cm^3^. The surface area of microvessels is 100–200 cm^2^ g^−1^ tissue (Crone, 1963; Gross et al., 1986), corresponding to a total surface area of 15–25 m^2^. In comparison, the surface area of the gut is 300–400 m^2^, the lung is about 100 m^2^, and the skin is about 2 m^2^. The microvessel density is about 500 m cm^−3^ (Kreczmanski et al., 2005, 2009), corresponding to a total microvessel length of about 600 km in the adult human brain (Zlokovic, 2005).

The human brain is comprised of about 100 billion neurons and a similar number of glial cells. Neurons, astrocytes, microglia, and pericytes account for almost 80% of the brain volume. The extracellular space occupies 15–30% of the brain volume (Nicholson, 2001) and the brain vasculature about 3% of the brain volume.(Nicholson, 2001) Capillaries in the brain may be as small as 7–10 μm in diameter and the average intercapillary distance is about 40 μm (Duvernoy et al., 1983; Nicholson, 2001). Consequently, the cell body of a neuron is typically about 10–20 μm from the nearest capillary (Schlageter et al., 1999).

Blood is supplied to the brain through four arteries, the internal carotid arteries and the vertebral arteries, which merge in the circle of Willis at the base of the brain (Hossmann, 2006). Each carotid artery supplies about 40% of the total blood flow to the brain. The flow rate to the brain is about 800 mL min^−1^ (Zlokovic, 2005), about 15–20% of the total blood flow from the heart. From the circle of Willis, intercerebral arteries, and pial arteries are distributed along the surface of the brain, from which arteries and arterioles penetrate into the brain parenchyma perpendicular to the brain surface, leading to the network of capillaries. In the human brain, capillaries form numerous connections before merging into venules and veins. Blood flow exits the brain through the jugular veins. While the arteries and arterioles are sheathed in one or more layers of smooth muscle cells, the capillaries are surrounded by pericytes and astrocytes.

The architecture of the vasculature can be described in terms of individual capillary segments between two junctions. The capillary length density ρ_*c*_ = *N*_*c*_L_*c*_/*V* where *N*_*c*_ is the number of segments of average length *L*_*c*_. Typical segment lengths in a mouse cortex are about 60 μm, with segment densities and junction densities of about 10,000 mm^−3^ (Heinzer et al., 2008). The tortuosity (τ) of a segment is given by τ = λ/*c*, where λ is the length of a segment and *c* is the chord length. Typical values for tortuosity in the mouse cortex are 1.2–1.3 (Heinzer et al., 2008). The number of segments connected at a junction *n* ≈ 3.5, indicating that a significant fraction of junctions are higher order than a simple bifurcation (*n* = 3).

Blood flow and heart rate are regulated by the autonomic nervous system, located in the medulla in the lower midbrain. The medulla receives sensory input from other brain regions and stimulates cardiovascular responses through nerve fibers that travel to the heart and blood vessels. Varicosities along the fibers are the sites for release of neurotransmitters. Autonomic and sensory nerve fibers are associated with the cerebral arteries, pial arteries, and arterioles in the brain, and release neurotransmitters such as norepinephrine (NE) and neuropeptide Y (NPY) that result in vessel constriction, and acetylcholine (Ach) and vasoactive intestinal polypeptide (VIP) that can dilate vessels. Regulation of brain capillaries by the autonomic nervous system has not been established, however, pericytes can dilate and contract in response to different neurotransmitters suggesting the possibility of autonomous signaling (Peppiatt et al., 2006; Fisher, 2009; Fernandez-Klett et al., 2010; Krueger and Bechmann, 2010).

Over the last 3 million years, from Australopithecus to Homo sapiens, the size of the human brain has increased from about 400 cm^3^ to about 1200 cm^3^ in modern humans (Aiello and Dunbar, 1993; Potts, 2011). This increase has not been continuous but has had several periods of rapid expansion. The expensive tissue hypothesis postulates that the increase in power consumption associated with increasing brain size must be balanced by a decrease in the power requirements in the liver and GI tract (Aiello and Wheeler, 1995). However, recent evidence suggests that these evolutionary increases in brain size are related to an increase in energy input, such as improved diet and availability of food, and changes in energy allocation, such as decreased energy costs associated with locomotion (Holliday, 1986; Roth and Dicke, 2005; Navarrete et al., 2011). During prenatal and early childhood development, the developing brain consumes 60% or more of the basal metabolism, and it has been argued that this is a fundamental limitation to brain size in humans (Snodgrass et al., 2009).

The microvasculature in the brain differs from other capillary networks in the human body, for example those in the lung, in two significant ways. First, the brain microvasculature tightly regulates transport into the brain. Second, the capillaries can exhibit significant plasticity in response to abnormal physiological conditions. For example, during ischemia capillaries can dilate to increase oxygen influx (Boero et al., 1999; Ito et al., 2003; Hauck et al., 2004).

The architecture of the brain microvasculature is very similar across species, indicating that the human brain microvasculature is not physically unique. However, an important question is whether the human blood-brain barrier is functionally different from other species. Evidence suggests that there may be significant biochemical differences, for example in the expression levels of transporters and pumps that make the human blood-brain barrier unique (Hammarlund-Udenaes et al., 2008). However, the evolutionary pressures that influenced these differences remains to be established. Elucidating these differences is key in studies of central nervous system diseases and in developing drug therapies.

### Maintaining brain homeostasis

The supply of metabolic nutrients to the human brain is achieved through a network of over 600 km of small capillaries, about 7 μm in diameter, such that each neuron is within 20 μm of a capillary. The drawback of this architecture is that the brain requires a tightly regulated local environment for cells to function. Since the brain microvasculature has a very large surface area (15–25 m^2^), maintaining homeostasis and preventing interference with signal generation and transmission in is a major challenge.

The blood-brain barrier is responsible for maintaining homeostasis of the brain by regulating the chemical environment, immune cell transport, and the entry of xenobiotics (Hawkins and Davis, 2005; Abbott et al., 2010). The concentrations of water, ions, amino acids, hormones, and neurotransmitters in the blood undergo fluctuations, particularly after eating or exercise. If such fluctuations were allowed to occur in the brain it would lead to local disruption of signal propagation and uncontrolled neural activity, and hence transport from the capillary lumen to the brain parenchyma must be tightly regulated. Immune cell transport (e.g., leukocytes) must also be regulated as the brain is contained in a fixed volume in the skull and an inflammatory response could lead to an increase in intercranial pressure or cerebral edema. Finally, the entry of toxins and pathogens, such as bacteria and viruses circulating in the blood, can lead to neuron cell death and hence must also be prevented (Begley and Brightman, 2003; Hawkins and Davis, 2005; Abbott et al., 2010).

**Figure 1:**
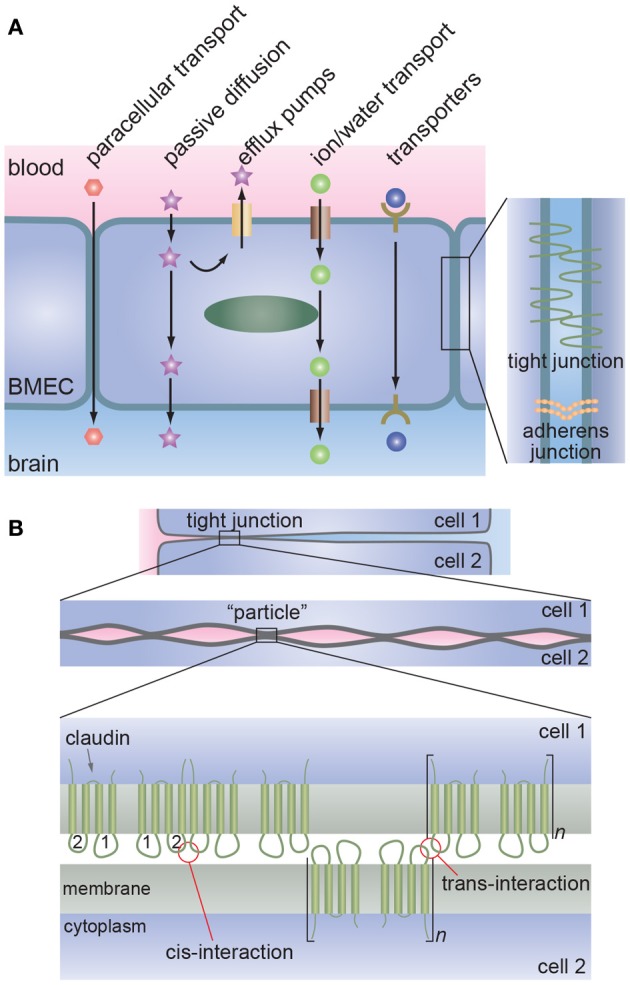
**The blood-brain barrier. (A)** Schematic illustration of transport across the brain microvascular endothelial cells (BMECs) that form the lumen of brain capillaries. Paracellular transport is severely restricted due to the formation of tight junctions between endothelial cells. Metabolic nutrients and other essential molecules are transported across the luminal and abluminal membranes by channels, pumps, or mediated transport systems. Small lipophilic molecules can passively diffuse across the lipid bilayer but, in many cases, are returned to the blood by efflux pumps. **(B)** Proposed molecular interactions at tight junctions. Lateral association of claudins (cis-interaction) results in the formation of oligomers whereas association of claudins on opposing membranes (trans-interaction) results in tight junction formation. Multiple regions of trans-interactions appear as particles in electron microscopy images.

The tight junctions formed by brain microvascular endothelial cells (BMECs) regulate paracellular transport whereas transcellular transport is regulated by specialized transporters, pumps, and receptors (Figure 1) (Chishty et al., 2001; Demeule et al., 2002; Hawkins et al., 2002; Ohtsuki and Terasaki, 2007; Ueno, 2009; Abbott et al., 2010; Hartz and Bauer, 2011).

### Circumventricular organs

While the blood-brain barrier maintains homeostasis, there are specialized regions of the brain that allow direct communication between the brain and the vascular system. In the circumventricular organs, located at the surface of the third and fourth ventricles, the blood-brain barrier is more permeable. Neurons and glial cells at these sites can sense changes in the concentration of various molecules, such as hormones, and secrete hormones, neurotransmitters, or cytokines into the circulation (Ganong, 2000; Duvernoy and Risold, 2007; Benarroch, 2011). These organs include: the neurohypophysis (posterior pituitary), the median eminence, the area postrema (vomiting center), the subfornical organ, and the vascular organ of the lamina terminalis.

## History

In 1885, Paul Ehrlich reported that various water-soluble dyes injected into the circulatory system did not stain the brain and spinal cord, and hypothesized that the CNS had a lower affinity for these dyes (Ribatti et al., 2006; Liddelow, 2011). In 1898, Biedl and Kraus showed that cholic acids (bile acids) that induce seizures and coma when injected into the brain, were not toxic when injected into the circulatory system (Ribatti et al., 2006). In 1900, Lewandowsky reported similar findings with potassium ferrocyanide and also concluded that there was limited permeation from the circulatory system into the brain (Ribatti et al., 2006), a phenomenon to which he ascribed the term *bluthirnschranke* (blood-brain barrier). Later, Ehrlich's student Edwin Goldmann showed that the water-soluble dye trypan blue (*MW* = 960.8) injected into cerebrospinal fluid readily stained central nervous tissue blue, contradicting Ehrlich's conclusion of a lower binding affinity of the central nervous system for these dyes, and supporting the hypothesis of limited permeation from the circulatory system into the brain (Ribatti et al., 2006). In 1967, Reese and Karnovsky used high resolution electron microscopy to demonstrate that horseradish peroxidase (HRP, 40 kDa) was prevented from entering the CNS by tight junctions (Bradbury, 1993). They showed that the tight junctions were continuous and concluded that the blood brain barrier existed at the level of the vascular endothelium.

## The neurovascular unit

### Introduction

**Figure 2:**
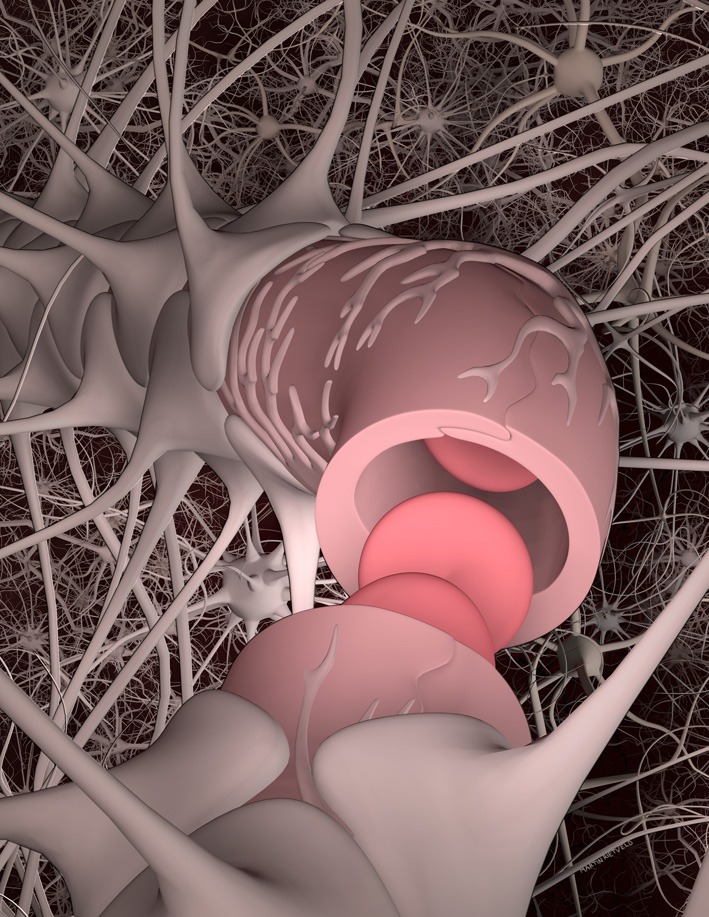
**The neurovascular unit**. The microvascular endothelial cells that form the lumen of brain capillaries are partially covered by pericytes and basement membrane, and almost completely surrounded by the end feet of astrocytes. Functional interactions between BMECs, astrocytes, pericytes, other glial cells, and neurons are key to regulating brain homeostasis. Blood flow is associated both biomechanical and biochemical signaling mediated by multiple cell types and soluble factors. The brain microvascular endothelial cells function in a cylindrical geometry with high curvature and experience shear stress resulting from blood flow. The BMECs and pericytes are surrounded by basement membrane consisting primarily of fibronectin, laminin 1, and collagen type IV. The extra-cellular matrix in the brain is based on hyaluronic acid.

Historically, the blood-brain barrier has been defined by the layer of endothelial cells that form the vessel/capillary walls. More recently, the concept of the neurovascular unit has been introduced to recognize that brain health depends on functional interactions between neurons and non-neuronal cells such as vascular cells (endothelial cells and pericytes) and glia (astrocytes, microglia, and oligodendroglia; Figure 2) (Hawkins and Davis, 2005; Abbott et al., 2010). This is a highly dynamic system in which cells transduce biochemical and biomechanical signals in complex microenvironments involving basement membrane and extracellular matrix. These non-neuronal cells are responsible for the physical, biochemical, and immune barriers of the CNS that regulate the microenvironment of the neurons which is key for signal transduction, remodeling, angiogenesis, and neurogenesis.

### Endothelial cells

The endothelial cells that line the microvasculature in the brain define the interface between the vascular system and the brain. These cells function as adaptive non-linear input/output devices where input from biomechanical and biochemical forces in the local microenvironment of the neurovascular unit influences cell phenotype as manifested by cell morphology, protein expression, gene expression, proliferation, transport, etc. (Dejana, 2004; Aird, 2005, 2007). In addition to biochemical and biomechanical input from the vascular system, numerous paracrine signaling pathways between microvascular endothelial cells and astrocytes and pericytes are responsible for maintenance of the blood-brain barrier (Aird, 2007; Abbott et al., 2010).

In the brain microvasculature, cell-cell junctions are key to maintaining the integrity of the brain microvasculature and regulating paracellular transport. Cell-cell adhesion is achieved through the formation of adherens junctions and tight junctions (Bazzoni and Dejana, 2004; Dejana, 2004; Aird, 2007). Both adherens junctions and tight junctions involve homophilic interactions between the extracellular domains of membrane proteins and are linked to the actin cytoskeleton via intracellular partners. Endothelial adherens junctions are formed by the extracellular domains of vascular endothelial cadherin (VE-cadherin) and are linked to the actin cytoskeleton inside the cell via proteins such as α-catenin, β-catenin, and vinculin (Bazzoni and Dejana, 2004; Dejana, 2004).

The tight junctions in the brain microvasculature prevent paracellular transport of most molecules and severely restrict transport of small ions. Therefore, transcellular transport is responsible for most molecular trafficking between the vascular system and the brain. Various methods for transient disruption of tight junctions have been explored for drug delivery, and local disruption of tight junctions is associated with many diseases of the central nervous system.

Tight junctions are formed between claudins (Nitta et al., 2003), although other proteins such as occludin are also present (Hawkins and Davis, 2005; Furuse and Tsukita, 2006). These tight junction membrane proteins are connected to the actin cytoskeleton via zona occludin (ZO) adaptor molecules (ZO-1 and ZO-2; Hawkins and Davis, 2005). The claudin family consists of more than 20 proteins that are essential for the formation of tight junctions. Claudin-5 is the isoform most commonly found in the BBB (Morita et al., 1999; Nitta et al., 2003; Hewitt et al., 2006), although claudin-1 and claudin-12 are also associated with tight junction formation (Wolburg and Lippoldt, 2002; Dejana, 2004; Abbott et al., 2006). Antibodies to claudin-5, occludin, and ZO-1 are commonly used as markers of tight junction formation in monolayers of BMECs (Cecchelli et al., 2007). Adherens junctions and tight junctions are structurally and functionally linked. For example, evidence suggests that VE-cadherin at adherens junctions upregulates the gene encoding the tight junction protein claudin-5 (Dejana, 2004; Taddei et al., 2008).

In high resolution electron microscope images, tight junctions appear as a series of discrete sites of apparent fusion near the apical surface (Reese and Karnovsky, 1967; Brightman, 1977). These sites are often described as strands of “particles” along the junction (Figure 2) (Tsukita and Furuse, 1999; Tsukita et al., 2001); the backbone of these strands is composed of claudins.

The claudins have a molecular weight of about 23 kDa and have four transmembrane segments, one intracellular loop, N- and C-terminal cytoplasmic domains, and two extra cellular loops (Furuse and Tsukita, 2006; Krause et al., 2009). The first extracellular loop ECL1 consists of about 50 amino acids whereas ECL2 consists of about 25 amino acids (Krause et al., 2009). Both of the extra-cellular loops (ECL1 and ECL2) of claudin-5 are thought to play a role in tight junction formation (Krause et al., 2009). Claudin-5 forms oligomers in one membrane via cis-interactions between ECL2s (Piontek et al., 2008). Trans-interactions between ECL2s on opposing membranes result in polymerization and formation of the particles observed in electron microscopy.(Piontek et al., 2008) Mutations of the two cysteines in claudin-5 have been shown to reduce barrier properties, suggesting that these two residues are important in tight junction formation (Wen et al., 2004).

The morphology of microvascular endothelial cells is dependent in part on biomechanical input from the vascular system. The shear stress associated with blood flow results in elongation and alignment of endothelial cells in the direction of flow (Caplan et al., 1974; Nerem et al., 1981; Ohashi and Sato, 2005; Aird, 2007). *In vitro* studies have confirmed that the elongation and alignment of endothelial cells in a 2D confluent monolayer increases with increasing shear stress (Levesque and Nerem, 1985; Malek and Izumo, 1996). In large vessels, there are many cells around the perimeter, however, in small capillaries endothelial cells can wrap around to form tight junctions with themselves, as well as their neighbors (Nag, 2003). Shear stress can also upregulate genes associated with junctional proteins and transporters (Cucullo et al., 2011).

The turnover of BMECs, measured as the mitotic index or the turnover time, is thought to be very low (Hobson and Denekamp, 1984; Ekstrand et al., 2008). However, most studies are based on extrapolation from relatively short intervals. The activation and increase in turnover of BMECs due to angiogenesis and vascular remodeling is also unknown. Similarly, the response of a capillary to endothelial cell apoptosis or disruption is not well-understood. Endothelial progenitor cells from bone marrow may be involved in repair of the blood-brain barrier however, the signaling processes involved in recruiting these cells and initiating differentiation are not known (Asahara et al., 2011).

### Astrocytes

Astrocytes are involved in multiple processes in the brain, including regulation of ion and water concentration, the clearance of neurotransmitters, proliferation of stem cells, control of the number of synapses, and maintenance of the BBB (Ullian et al., 2001; Lee et al., 2003; Volterra and Meldolesi, 2005; Abbott et al., 2006; Fiacco et al., 2009; Freeman, 2010; Halassa and Haydon, 2010). Morphologically, astrocytes are usually star shaped with many processes or protrusions emanating from the cell body, with an overall diameter of about 140 μm in the human brain (Oberheim et al., 2009). Astrocytes interact with microvascular endothelial cells through the end-feet of the protrusions that wrap around the capillary (Abbott, 2002; Abbott et al., 2006). Brain capillaries are often almost completely surrounded by astrocytic end-feet and one astrocyte may contact multiple capillaries (Oberheim et al., 2009). Astrocytes form contacts with microvessels and the synapses between neurons, and play an important role in matching oxygen and glucose transport to neural activity through regulation of local blood flow (Zonta et al., 2003; Iadecola, 2004; Takano et al., 2006; Iadecola and Nedergaard, 2007). Evidence suggests that intracellular Ca^2+^ is involved in blood flow regulation since neuronal stimulation results in an elevation of intracellular Ca^2+^ concentration in astrocyte end-feet (Iadecola, 2004).

Astrocytes participate in the formation of the BBB by enhancing tight junction formation, modulating the expression and polarization of transporters, and promoting specialized enzyme systems (debault and Cancilla, 1980; Janzer and Raff, 1987; Abbott, 2002; Lee et al., 2003; Haseloff et al., 2005; Abbott et al., 2006). Several astrocyte derived factors, including glial-derived neurotrophic factor (GDNF), basic fibroblast growth factor (BFGF), and angiopoetin-1 (ANG-1) are known to induce blood-brain barrier characteristics in endothelial cells (Haseloff et al., 2005; Abbott et al., 2006).

### Pericytes

Pericytes wrap around microvessels and capillaries in the brain (Sims, 1986; Fisher, 2009; Attwell et al., 2010; Krueger and Bechmann, 2010; Dalkara et al., 2011; Winkler et al., 2011) and communicate with endothelial cells, astrocytes, and neurons in the neurovascular unit (Bonkowski et al., 2011). Morphologically, pericytes tend to be aligned with the vessel axis and extend protrusions that wrap around the capillaries (Bonkowski et al., 2011). A thin layer of basement membrane separates pericytes from endothelial cells, and from surrounding astrocyte end-feet. The ratio of pericytes to endothelial cells is typically around 1:3 (Shepro and Morel, 1993). Direct peg-and-socket contacts that span the intervening basement membrane and gap junctions with endothelial cells initiate multiple signaling pathways (Bonkowski et al., 2011). For example, platelet-derived growth factor B (PDGF-B) on endothelial cells binds with the corresponding receptor (PDGFR-B) on pericytes, regulating recruitment of pericytes as well as their proliferation (Bell et al., 2010; Dalkara et al., 2011; Winkler et al., 2011).

Pericytes are contractile, with actin stress fibers throughout the cell body, and contribute to the regulation of blood flow by controlling capillary diameter (Peppiatt et al., 2006; Hamilton et al., 2010; Dalkara et al., 2011). In cell culture, pericytes are usually identified by α-smooth muscle actin, which is not expressed in endothelial cells, although expression can be heterogeneous.(Dalkara et al., 2011) Pericytes do not express GFAP, expressed by astrocytes, or vWF, expressed by ECs.

Studies in mice have shown that perictyes are recruited to nascent capillaries during development (Daneman et al., 2009, 2010) and are key for development of the BBB and regulating transport across the BBB (Armulik et al., 2005, 2010; Daneman et al., 2009, 2010; Kim et al., 2009) Indeed, pericyte loss leads to locally reduced cerebral blood flow and breakdown of the blood-brain barrier (Armulik et al., 2010; Bell et al., 2010).

Pericytes are able to either enhance or impair blood brain barrier function in *in vitro* models depending on their state of differentiation. Pericytes differentiated with TGFβ are α-actin positive and have been found to decrease transendothelial electrical resistance (TEER), while pericytes differentiated with BFGF are α-actin negative and increase TEER above controls (Thanabalasundaram et al., 2011).

### Cell lines for research

A major challenge for the development of *in vitro* models of the BBB is the availability of appropriate cell lines, particularly BMECs. An *in vitro* model of the human BBB should exhibit restricted paracellular transport (TEER = 1 kΩcm^2^, *P*_sucrose_ < 10^−7^ cm s^−1^), BMECs with the morphology and characteristics typical of the BBB, expression of BBB-specific markers and transporters, and be readily available, convenient to use, and reproducible (Reichel et al., 2003). While primary human BMECs are often considered preferable for *in vitro* models, the difficulties in harvesting and purification of these cells can significantly limit accessibility and reliability (Stins et al., 1997; Bernas et al., 2010). In general, primary cells are used only at very low passage numbers to avoid down-regulation of BBB characteristics (Reichel et al., 2003). In contrast, currently available cell lines can overcome limitations associated with accessibility and convenience, but do not exhibit all of the required features of the human BBB (Sloan et al., 2012). Nevertheless, specific cell lines may recapitulate properties that are necessary for some physiological, pathological, or pharmacological applications. Common sources for animal BMECs include rodent, bovine, and porcine brain cortices. Primary astrocytes and pericytes can also be extracted from the brain cortex (Siddharthan et al., 2007). Advances in stem cell engineering suggest that differentiation of stem cells to BMECs (Lippmann et al., 2012) and astrocytes (Krencik et al., 2011) may ultimately solve the problem of limited cell lines.

### Basal lamina and ECM

The basement membrane surrounding the endothelial cells and pericytes is comprised of fibronectin, laminin (411, 421, and 511) (Aumailley et al., 2005), and collagen type IV (Tilling et al., 1998, 2002; Hartmann et al., 2007). The thickness of the basement membrane, determined from electron microscope images, is about 100 nm (Nag, 2003). Endothelial cell monolayers on fibronectin, laminin 1, and collagen type IV show enhanced TEER, suggesting a role for the basement membrane in enhancing the formation of tight junctions (Tilling et al., 1998, 2002; Hartmann et al., 2007).

The extracellular matrix in the brain is composed of four main components: hyaluronic acid (HA), lecticans, hyaluronan and proteoglycan link proteins (HAPLNs), and tenascins (Zimmermann and Dours-Zimmermann, 2008). Common ECM proteins such as fibronectin and collagen type I are not present in the brain (Sanes, 1989). HA is a long unbranched polysaccharide with negatively charged disaccharide repeat units, and is unique amongst the glycosaminoglycans (GAGs) in that it is non-sulfated (Laurent and Fraser, 1992; Toole, 2004; Zimmermann and Dours-Zimmermann, 2008; Ananthanarayanan et al., 2011). HA is synthesized by hyaluronan synthases at the inner surface of the plasma membrane, and can have a molecular weight as high as 10^7^ Da (Zimmermann and Dours-Zimmermann, 2008). HA can interact with cells through binding to cell surface receptors, such as CD44 and RHAMM (Turley et al., 2002). The lecticans are a family of chondroitin sulfate proteoglycans with an HA binding domain and include aggrecan, versican, neurocan, and brevican (Yamaguchi, 2000). The tenascins (Tns) are large multimeric glycoproteins. Tn-C and Tn-R are thought to bind to multiple lecticans and link proteins thereby crosslinking the HA and forming a 3D network (Zimmermann and Dours-Zimmermann, 2008).

The interstitial fluid in the brain is similar in composition to blood plasma, however, it has lower K^+^ and Ca^2+^ concentrations but higher Mg^2+^ concentration. In addition, the interstitial fluid has a lower protein content than plasma.

### Extracellular space (ECS)

The ECS is the region between cells in the brain and provides the main pathway for transport between capillaries and neurons and other cells in the brain. Although most neurons are within 10–20 μm of a capillary, transport in the extracellular space is usually much faster than transport across the BBB and hence is particularly important for local penetration of a solute. The ECS consists of the hyaluronan-based ECM and a fluid phase, and is characterized by a volume fraction α (= *V*_ECS_/*V*_brain_) of 0.15–0.30 (Sykova and Nicholson, 2008). The fluid phase serves as a reservoir for extracellular ions necessary for generating action potentials, a medium for transporting molecules such as neurotransmitters involved in signaling, and for transporting essential molecules between microvessels and cells in the brain. The extracellular volume fluctuates during normal brain function and decreases during development and aging (Sykova and Nicholson, 2008; Kroeger et al., 2010).

The geometry of the extracellular space has been modeled as an interconnected network of sub-100 nm pores resembling sheets and tunnels (Sykova and Nicholson, 2008; Kinney et al., 2013). Sheets represent regions where the plasma membranes of two cells are in close proximity, similar to two parallel plates, and tunnels correspond to approximately cylindrical channels. The geometry of the network of pores in the ECS restricts diffusion in the brain compared to free diffusion, and is characterized by the tortuosity, λ which is defined by the ratio (Δ_free_/Δ_brain_)^1/2^ where *D*_free_ is the diffusion coefficient in solution and *D*_brain_ is the diffusion coefficient in the brain (Nicholson, 2001). The tortuosity takes into account the fact that molecules must detour around cells during transport.

Results from tracer experiments suggest a value of λ ≈ 1.6 in the rat brain. Since the diffusion coefficient for K^+^ and Cl^−^ ions in aqueous solution is around 2 × 10^−5^ cm^2^ s^−1^, a tortuosity of 1.6 implies a diffusion coefficient in the brain of around 0.8 × 10^−5^ cm^2^ s^−1^. Analysis of *in vivo* experiments using probes with different hydrodynamic radii suggests effective pore dimensions of about 40 nm between parallel plates and about 60 nm for cylindrical channels (Thorne et al., 2005; Thorne and Nicholson, 2006). As a result, the transport of molecules or particles approaching these dimensions will be limited due to steric hindrance and drag by the pore walls (Thorne et al., 2005; Thorne and Nicholson, 2006).

While global biophysical parameters such as ECS volume fraction, tortuosity, and effective pore size have been estimated, the details of the physico-chemical properties that control transport between capillaries and neurons and other cells in the brain remain to be established. Transport in the ECS may also be modulated by dead-end branches in the ECS network, transient binding with the ECM in the extracellular space, transient binding with cell membranes, or cell uptake (Sykova and Nicholson, 2008).

### Shear stress

Blood pressure exerts a force normal to a vessel wall that imposes a circumferential stress on the vessel, whereas blood flow results in a frictional drag, or shear stress, parallel to the endothelium in the direction of blood flow. These stresses play an important role in regulating endothelial cell morphology and function, and in mediating a wide range of signaling and transport processes between the vascular system and surrounding tissue (Chien, 2007; Hahn and Schwartz, 2009; Johnson et al., 2011; Conway and Schwartz, 2012). These stresses are also thought to play an important role in regulation of the blood-brain barrier (Krizanac-Bengez et al., 2004; Neuwelt et al., 2008, 2011; Tarbell, 2010; Cucullo et al., 2011).

For an ideal Newtonian fluid (incompressible), the shear stress τ in a straight cylindrical vessel under constant laminar flow is given by the Poiseuille equation: τ = 4μQ/πr^3^ where μ is the dynamic viscosity, *Q* is the volumetric flow rate, and *r* is the radius of the lumen. Therefore, the magnitude of the shear stress on the endothelium is proportional to the flow rate and viscosity, and inversely proportional to *r*^3^. Consequently, endothelial cells in vessels with high flow rate and small diameter are exposed to large shear stress.

The viscosity of blood is about 4 cP (0.004 Pa·s), significantly larger than the viscosity of water of 0.7 cP (0.0007 Pa·s) at 37°C, primarily due to the presence of red blood cells. Typical time averaged values of shear stress are 4–30 dynes cm^−2^ in the arterial circulation and 1–4 dynes cm^−2^ in the venous circulation (Turitto, 1982; Kamiya et al., 1984; Papaioannou and Stefanadis, 2005; Koutsiaris et al., 2007; Dolan et al., 2013). The flow rate in capillaries is typically from 6 to 12 nL min^−1^ corresponding to a shear stress of 10–20 dynes cm^−2^ for a capillary 10 μm in diameter (taking μ = 1 cP or 0.001 Pa s; Kamiya et al., 1984).

As described above, the viscosity of bulk blood is around 4 cP, significantly higher than the viscosity of water, due in large part to the density of red blood cells. A complication arises in small capillaries since cells tend to avoid the vessel walls resulting in a cell-free layer within about 3 μm of the surface that has a viscosity close to that of water, a phenomenon known as the Fahraeus—Lindqvist effect (Fahraeus and Lindqvist, 1931). For large diameter vessels, this effect is negligible and the effective viscosity is close to the bulk viscosity of blood. However, for smaller diameter vessels, the cell-free layer can become a significant fraction of the cross-sectional area resulting in a decrease in effective viscosity.

## Transport across the BBB

### Introduction

The barrier function of the BBB is critical for regulating transport to the brain, but also represents a significant roadblock in delivering drugs to the brain (Pardridge, 2003, 2005, 2006, 2008, 2010; Hawkins and Davis, 2005; Ohtsuki and Terasaki, 2007). Only very few CNS disorders, such as depression, schizophrenia, chronic pain, and epilepsy, are currently treatable with small molecule drug therapy. The BBB is the major roadblock in developing therapies for CNS diseases including neurodegenerative diseases, cerebrovascular diseases, and brain cancer. Therefore, methods to study the transport of drugs and other molecules across the blood-brain barrier are key to understanding how the BBB regulates transport and will be invaluable for drug discovery and the treatment of CNS diseases (Cecchelli et al., 2007; Kuhnline Sloan et al., 2012).

**Figure 3:**
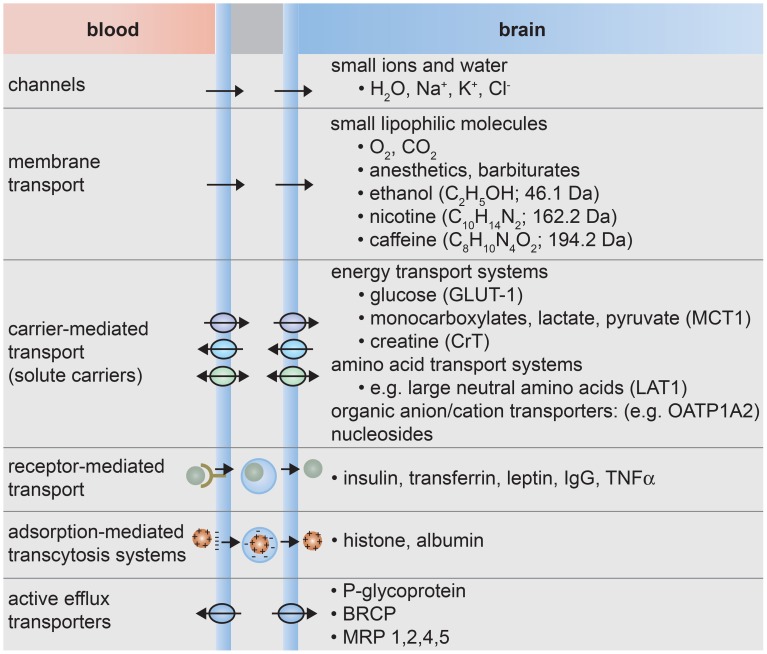
**Transport systems at the blood-brain barrier**. (1) Small ions and water molecules can cross the blood-brain barrier through ion channels. (2) Small lipophilic molecules that are soluble in the hydrophobic core of the cell membrane can be transported passively across the cell. (3) Essential polar molecules that cannot diffuse through the cell membrane are shuttled across the cell membranes by carrier-mediated transport. These solute carriers may be directional, in or out of the cell, or bidirectional. Other molecules can be actively transported across endothelial cell membranes by carrier-mediated transporters, receptor-mediated transporters, adsorption-mediated transcytosis, or efflux pumps.

The formation of tight junctions effectively eliminates paracellular transport across the blood-brain barrier (de Boer et al., 2003; Cecchelli et al., 2007; Abbott et al., 2010; Giacomini et al., 2010). Transcellular transport can occur through various mechanisms (Lee et al., 2001; de Boer et al., 2003; Cecchelli et al., 2007; Ohtsuki and Terasaki, 2007; Ueno, 2009; Abbott et al., 2010; Giacomini et al., 2010), as illustrated in Figure 3. Small lipophilic molecules can enter the brain via passive diffusion across the luminal and abluminal cell membranes. To regulate passive transport into the brain, efflux pumps return many unwanted molecules back to the circulatory system. Small polar molecules, such as glucose, amino acids, organic anions and cations, and nucleosides, can cross the blood-brain barrier by carrier-mediated transport. These solute carriers may be specific to one molecule or multi-specific to several molecules. Large solutes, such as proteins and peptides, are transported across the BBB by receptor-mediated or adsorption-mediated endocytic transport. Highlighting the important role of transport, it has been estimated that 10–15% of all proteins in the neurovascular unit are transporters (Enerson and Drewes, 2006). As a result of this regulated transport, there can be large differences in the concentration of amino acids and proteins while differences the concentration of ions in the blood and cerebral spinal fluid are relatively small (Abbott et al., 2006).

Passive transport is a way to bypass the array of substrate specific transport systems that are designed to regulate transport across the blood-brain barrier. In general, passive transcellular transport is limited to small molecules that have a combination of sufficient hydrophilicity to be soluble in water and sufficient lipophilicity to be soluble in the hydrophobic core of the lipid bilayer. Small gaseous molecules such as O_2_ and CO_2_ can diffuse through the cell membrane, as well as small molecules such as barbiturates, ethanol, and caffeine. Almost no large molecules and 98% of all small molecules do not cross the BBB.(Pardridge, 1998, 2010) In general, molecules that passively diffuse across the BBB have a *MW* < 500 Da, log *P*_*oct*_ in the range 2–4, and the number of hydrogen bond donors is less than 5 (Avdeef, 2001; Lipinski et al., 2001). Many molecules that cross the membrane by passive transport are subsequently transported back to the vascular system by efflux pumps.

The details of transport from a capillary into the brain remain poorly understood. As described above, BMECs are surrounded by pericytes that extend processes over the capillary surface, a 50–100 nm thick basement membrane, and astrocyte end-feet that almost completely surround the outside of the capillaries. Transport across an endothelial cell in a capillary may not be radially symmetric since the cell thickness, and hence diffusion length, is dependent on position. From transmission electron microscope images of rat brain capillaries, the endothelial cell thickness ranges from about 0.2 μm away from the nucleus to about 0.9 μm in the vicinity of the nucleus (Farkas and Luiten, 2001; Nicaise et al., 2009). Once transported across the endothelium, a molecule enters the basement membrane where it can be transported into a pericyte or astrocyte, or can diffuse laterally to a gap between astrocyte end-feet and into the extracellular space. To predict the spatial and temporal distribution of a molecule in the brain will require detailed characterization of the transport properties of the cellular and matrix components of the neurovascular unit, along with an understanding of how these properties change with time, for example during development, aging, and disease.

### Lipophilicity

Lipophilicity is the affinity of a molecule for a lipophilic environment (McNaught and Wilkinson, 1997). The partition coefficient *P* is the ratio of the concentration of the molecule in a solvent such as octanol to the concentration in water (*P*_oct_; Avdeef, 2001; Waterhouse, 2003). The solvent is selected to mimic the hydrophobic environment of the core of a lipid bilayer. Explicitly, *P* is defined for all neutral species and is pH independent. If the molecule can be ionized then the lipophilicity is determined by the distribution coefficient D which takes into account ionized species. Depending on the pKa, the distribution coefficient will show a pH-independent regime where the molecule is neutral and a pH dependent regime where the molecule is ionized. In general, ionization results in increased solubility but decreased partitioning to octanol.

The lipid composition of the membrane of human BMECs is ~33% phosphatidyl choline (PC), 25% phosphatidyl ethanolamine (PE), 17% sphyngomyelin (Sph), 11% phosphatidyl serine (PS), 4.8% phosphatidyl inositol (PI; Siakotos et al., 1969; Tewes and Galla, 2001).

### *In vitro* measurements

#### The transwell assay

**Figure 4:**
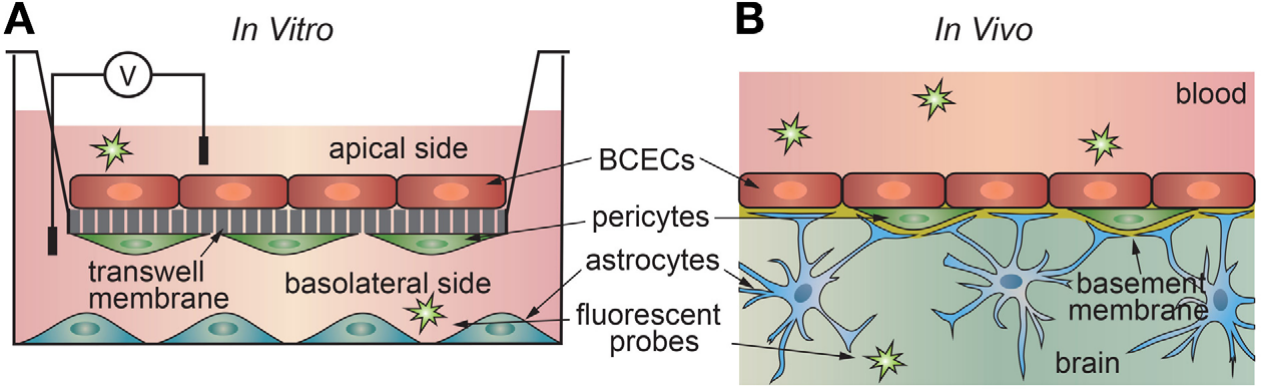
**Schematic illustration of (a) *in vitro* and (b) *in vivo* transport measurements. (A)** In the 2D transwell assay, a monolayer of cells is formed on a porous membrane separating two compartments. Astrocytes and/or pericytes may be seed on the opposite side of the membrane or in the output chamber. **(B)** *In vivo* studies, a solute is injected into the blood of an animal model, and the penetration into the brain measured using a suitable chemical detection assay or imaging technique.

The development of an *in vitro* platform to study transport across the blood-brain barrier has proven challenging. This is not surprising, as brain capillary endothelial cells transduce signals from surrounding astrocytes, pericytes, and from the vascular system. *In vitro* transport measurements are usually performed using a transwell assay where a confluent monolayer of endothelial cells is formed on a porous support separating two chambers (Figure 4). The permeability can be determined from the transport of a drug or fluorescent probe from the donor side to the acceptor side. Alternatively, ion transport can be characterized in terms of the electrical impedance of the monolayer.

The transwell assay is widely used to study absorption of orally administered molecules in the intestine (Artursson, 1991; Artursson et al., 2001; Stenberg et al., 2001; Hubatsch et al., 2007). Caco-2 cells of passage 30–45 are plated on polymer membranes with 1 μm pores and cultured for 20–23 days to confluence. Confluence is confirmed by a resistance measurement of over 200 Ωcm^2^. After confluence is reached, permeability measurements can be made across the monolayer. A permeability of >10^−6^ cm s^−1^ is correlated with 100% oral absorption, whereas a permeability of <10^−7^ cm s^−1^ is correlated with less than 1% oral absorption. Permeabilities for common drugs include 5.3 × 10^−5^ cm s^−1^ for ibuprofen, 2.0 × 10^−6^ cm s^−1^ for benzyl penicillins, and 1.6 × 10^−7^ cm s^−1^ for doxorubicin (Yee, 1997). Caco-2 cells are also widely used for determining whether a substance is a P-gp substrate by measuring the bidirectional permeability (Balimane et al., 2006).

2D models of the blood-brain barrier for transport studies historically utilize monolayers of type II Madin-Darby canine kidney (MDR1-MDCK) cells, genetically engineered to express the Pg-p pump. Other cell types used for the transwell assay are primary BMECs isolated from human or animal brain tissue. These cells are usually plated on transwell membranes coated with rat tail collagen I or basement membrane proteins such as collagen IV, fibronectin, laminin (Tilling et al., 1998). BMECs are often co-cultured with astrocytes and/or pericytes to induce blood-brain barrier properties. Astrocytes are commonly cultured in the lower compartment of the transwell dish, either on the opposite side of the membrane from the endothelial cells or on the bottom of the dish to provide secreted factors (Siddharthan et al., 2007). Astrocyte-conditioned media is also commonly used in transwell systems, as the soluble factors secreted by astrocytes, such as bFGF and GDNF, have been shown to increase tight junction properties (Abbott et al., 2006). The influence of pericytes on transport in the transwell assay is not well-understood and may be dependent on their stage of differentiation (Thanabalasundaram et al., 2011). Tri-culture models with endothelial cells plated on a transwell membrane, either astrocytes or pericytes on the opposite side of the membrane, and the third cell type plated on the bottom of the dish have been found to improve blood-brain barrier properties compared to co-culture (Nakagawa et al., 2009; Hatherell et al., 2011).

#### Transendothelial electrical resistance (TEER)

The first *in vivo* transendothelial electrical resistance measurements were performed on frog brain microvessels in a two-electrode configuration with one electrode inserted into the vessel and the other used to measure the voltage drop as a function of distance along the vessel (Crone and Olesen, 1982). The voltage drop along a cylindrical vessel can be related to the TEER:

(1) $$V\left(\text{x}\right)=V\left(0\right)\text{exp}\left(-\frac{x}{\lambda }\right)$$

where *V*(*x*) is the voltage at distance *x*, and λ is the characteristic length which depends on the vessel radius and the resistivity of blood. The transendothelial resistance *R*_*m*_ of the endothelial cells defining the lumen of the vessel is then determined from:

(2) $$R_{m}=2\pi aR_{i}\lambda$$

where *R*_*i*_ is the internal resistance of the capillary (Ω cm^−1^) and *a* is the vessel diameter (cm). The internal resistance is given by:

(3) $$R_{i}=\frac{\rho_{i}}{\pi a^{2}}$$

where ρ_*i*_ is the resistivity of blood (Ωcm). Blood resistivity is exponentially dependent on the hematocrit, with a typical value of around 120 Ωcm corresponding to a 40% hematocrit. Blood plasma has a resistivity of around 50 Ωcm (see Supplementary Information). Note that the resistance is normalized to unit area of the endothelium and has units of Ωcm^2^.

From these experiments the average resistance was determined to be 1840 Ωcm^2^ (Crone and Olesen, 1982). Subsequent experiments with rat brain surface microvessels determined the average resistance of venous microvessels to be 800 Ωcm^2^, and the average resistance of arterial microvessels to be 2000 Ωcm^2^, with an overall average of 1500 Ωcm^2^ (Butt and Jones, 1992). TEER measurements have been widely used to characterize tight junctions (Madara, 1998; Franke et al., 1999; Gumbleton and Audus, 2001; Reichel et al., 2003; Deli et al., 2005; Shen et al., 2011).

TEER measurements using the transwell assay generally result in resistances much lower than the values of 1500–2000 Ωcm^2^ reported for *in vivo* measurements. Values in excess of 150–200 Ωcm^2^ are generally considered suitable for studying solute and drug transport (Reichel et al., 2003). There are two contributing factors to this difference. First, the transwell assay does not recapitulate all of the physical and biological features of the BBB, resulting in the formation of cell-cell junctions that are not quite as effective in restricting paracellular transport. Second, there is usually a short circuit path due to incomplete monolayer formation or poor adhesion to the walls of the transwell support. In the transwell assay, if we consider a short circuit resistance in parallel with the endothelial resistance then the measured resistance is given by:

(4) $$R_{\text{meas}}=R_{S}+\frac{1}{{\left(\frac{R_{\text{SC}}}{1-f_{c}}\right)}^{-1}+{\left(\frac{R_{m}}{f_{c}}\right)}^{-1}}$$

where *R*_*s*_ is the solution resistance, *R*_sc_ is the short circuit resistance, *R*_*m*_ is the endothelial cell resistance, and *f*_*c*_ is the fraction of the transwell surface covered with a confluent monolayer of endothelial cells. Note that when *f*_*c*_ → 1, *R*_meas_ ≈ *R*_*m*_ as long as *R*_*m*_ » *R*_*s*_. Taking *R*_*s*_ = 2 Ωcm^2^, *R*_sc_ = 5 Ωcm^2^, and *R*_*m*_ = 1500 Ωcm^2^, the typical range of resistances for *in vitro* measurements correspond to a coverage fraction from 0.90 to 0.98. The problem associated with a short circuit path at the perimeter of the TEER device can be overcome by plating cells on microfabricated electrodes. The disadvantage of using microfabricated electrodes is that there is no solution reservoir underneath the cell monolayer making it very difficult to perform transport measurements.

While the relatively low TEER values usually obtained in transwell experiments (150–200 Ωcm^2^) makes it difficult to compare experiments quantitatively to *in vivo* conditions, it still allows qualitative assessment of conditions that induce BBB properties. For example, the important role of astrocytes in inducing BBB properties is demonstrated by the increase in TEER values observed by co-culture of BCECs with astrocytes or astrocyte-conditioned media (debault and Cancilla, 1980; Abbott, 2002). Similarly, the increase in TEER observed in tri-culture models provides additional evidence for the role of pericytes in the induction of BBB properties.

### Permeability

**Figure 5:**
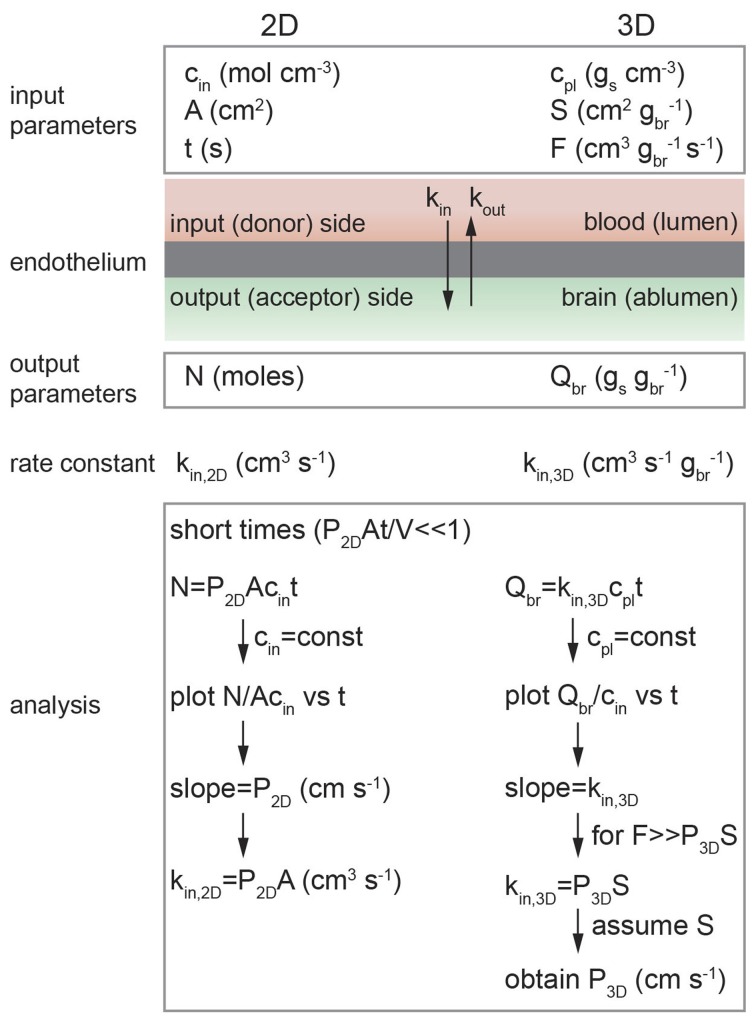
**Schematic illustration of analysis of diffusion transport in 2D and 3D**. In 2D: *c*_in_ is the solute concentration on the input side in a transwell assay, *A* is the area of the cell monolayer (cm^2^), *N* is the number of moles of solute measured on the output side in volume *V* (cm^3^), and *k*_in, 2D_ is the 2D rate constant (cm^3^ s^−1^). As long as *c*_in_ = constant then *k*_in, 2D_ = *P*_2D_*A*. In 3D: *c*_pl_ is the solute concentration in plasma (g_s_ cm^−3^), *S* is the normalized surface area of the lumen (cm^2^ g_br_^−1^), and *F* is the normalized flow rate (cm^3^ g_br_^−1^ s^−1^), *Q*_br_ is the amount of solute transported to the brain (g_s_ g_br_^−1^), and *k*_in, 3D_ is the 3D rate constant (cm s^−1^ g_br_^−1^).

The rate of transport of a solute across a barrier is characterized by the permeability, which is defined as the flux through unit area under unit concentration gradient and has units of cm s^−1^. It is implicitly assumed that there is no hydrostatic or osmotic pressure difference across the barrier (Kedem and Katchalsky, 1958). In the analysis of transport across the blood-brain barrier, the endothelium is generally considered a black box with first order rate constants *k*_in_ and *k*_out_ where *k*_in_ describes transport of a solute into the brain and *k*_out_ describes the reverse process (see Figure 5).

The flux (moles s^−1^) of a solute across a volume element is given by:

(5) $$J=\frac{dN}{dt}=k_{\text{in}}c_{\text{in}}-k_{\text{out}}c_{\text{out}}$$

where *N* is the number of moles transported across the volume element, *c*_in_ is the concentration (mol cm^−3^) on the input side and *c*_out_ is the concentration on the output side. In all experiments it is implicitly assumed that paracellular transport across the tight junctions is negligible. For the case where transport is dominated by passive diffusion across the cell membranes then *k*_in_ = *k*_out_ assuming negligible difference in lipid composition between the luminal and abluminal membranes. However, if a solute is a substrate for a transporter, such as an efflux pump then *k*_in_ ≠ *k*_out_.

The flux may be normalized in different ways, depending on the type of experiment. For example, the *in vitro* transwell assay is a 2D assay where the input and output compartments are separated by a monolayer of endothelial cells. In contrast, *in vivo* brain perfusion is a 3D assay. The difficulties in performing *in vivo* transport measurements can make comparison of 2D and 3D measurements somewhat confusing, for example, the *in vitro* (2D) rate constant *k*_in, 2D_ is normalized to unit area whereas the *in vivo* (3D) rate constant *k*_in, 3D_ is usually normalized to unit mass.

### 2D transport

*In vitro* transport studies are typically performed using a 2D transwell assay where a confluent monolayer of endothelial cells on a porous support is located between input and output chambers (Figure 4) (Siflinger-Birnboim et al., 1987; Karlsson and Artursson, 1991; Artursson, 1991; Adson et al., 1994; Cecchelli et al., 1999; Youdim et al., 2003; Deli et al., 2005). A solute, typically radio-labeled or fluorescently-labeled, is introduced into the input chamber and the amount accumulated in the output chamber is measured as a function of time, typically over a period of 1–2 h (Bowman et al., 1983; Audus and Borchardt, 1986; Shah et al., 1989; Karlsson and Artursson, 1991; Artursson, 1991; Freed et al., 2001; Chappa et al., 2006; Summerfield et al., 2007). The concentration of solute on the input side *c*_in_ (mol cm^−3^) and the area *A* (cm^2^) of the monolayer are the input parameters, and the concentration of solute on the output side is measured as a function of time. Note that *c*_out_ = *N/V* where *N* is the number of moles of solute and *V* (cm^3^) is the fluid volume in the output chamber.

**Figure 6:**
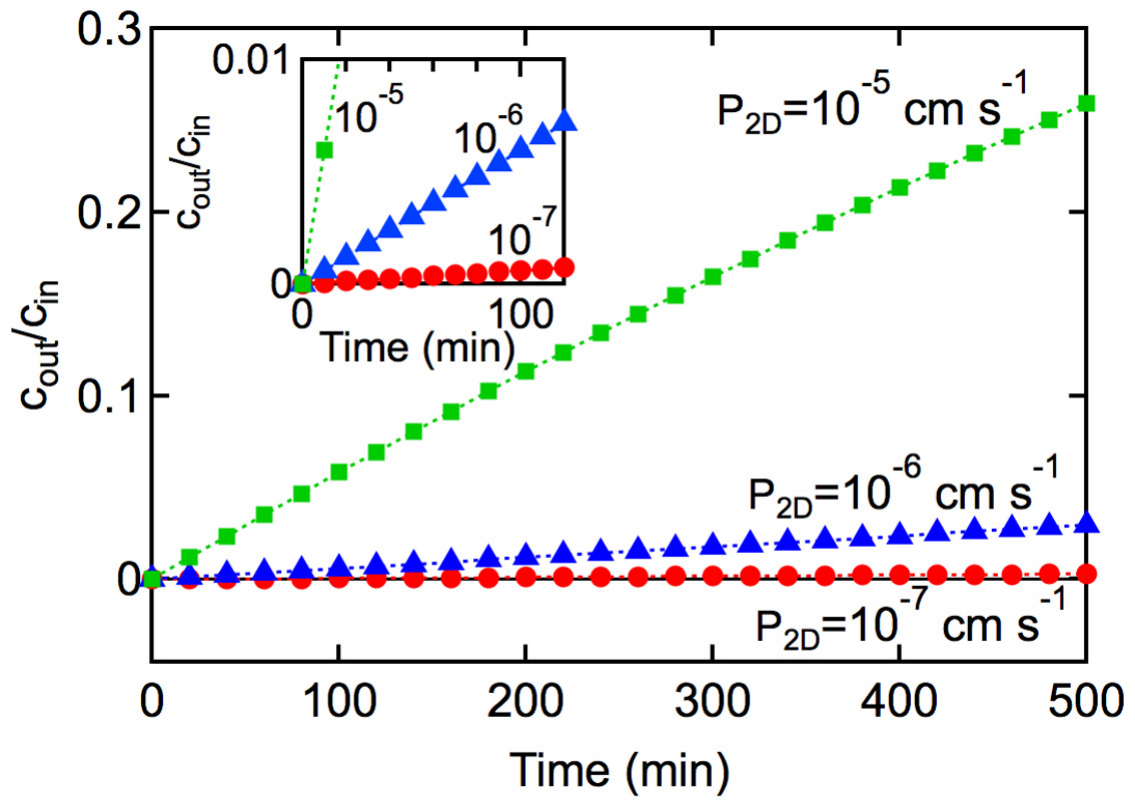
**Kinetics of solute transport across a 2D monolayer**. *c*_out_/*c*_in_ is plotted as a function of time *t*, with *A* = 1 cm^2^, and *V* = 1 cm^3^ for (■) *P*_2D_ = 10^−5^ cm s^−1^, and (▲) 10^−6^ cm s^−1^, and (•) P_2D_ = 10^−7^ cm s^−1^, At short times (inset) where *P*_2D_*At*/*V* « 1, the slope is *P*_2D_*A*/*V* and the rate constant can be obtained from *k*_in,2D_ = *P*_2D_*A*.

Integrating Fick's first law and recognizing that *k*_in, 2D_ = *P*_2D_*A* where *P*_2D_ is the permeability (cm s^−1^), we obtain:

(6) $$N(t)=Vc_{\text{in}}\left(1-\exp\left(-\frac{P_{\text{2D}}A}{V}t\right)\right)$$

(see Figure 6 and Supplementary Information) (Kedem and Katchalsky, 1958; Siflinger-Birnboim et al., 1987; Dawson, 1991; Tran et al., 2004)At short times where *P*_2D_*At* « *V*, the exponential term can be linearized and hence:

(7) $$N(t)=P_{\text{2D}}Ac_{\text{in}}t$$

In the derivation of Equation 7 it is assumed that: (1) the concentration of solute in the input chamber (*c*_in_) is approximately constant, (2) transport from the output chamber to the input chamber can be neglected (*k*_out_*c*_out_ → 0), and (3) transport is dominated by passive diffusion across the cell membrane (see Supplementary Information). Experimentally, the permeability can be obtained from the slope of a plot of *N*(*t*)/*Ac*_in_ vs. time at short times where *P*_2D_*At*/*V* « 1. Alternatively, *c*_out_/*c*_in_ can be plotted against *t* where the slope is *P*_2D_*A*/*V*. The rate constant can then be obtained from *k*_in, 2D_ = *P*_2D_*A*.

Experimentally, transport of a solute from the input chamber to the output chamber involves transport across the aqueous boundary layer above the cell monolayer, transport across the cell monolayer, and transport through the porous membrane. These steps are in series and hence we can write:

(8) $$\frac{1}{P_{2\text{D}}}=\frac{1}{P_{m}}+\frac{1}{P_{f}}+\frac{1}{P_{S}}$$

where *P*_*m*_ is the permeability coefficient of the cell monolayer, *P*_*f*_ is the permeability coefficient of the transwell membrane, and *P*_*S*_ is the permeability of the boundary layer above the cell monolayer (Barry and Diamond, 1984; Karlsson and Artursson, 1991; Artursson, 1991; Adson et al., 1994; Avdeef et al., 2005). To ensure that the measured permeability coefficient *P*_2D_ = *P*_*m*_ requires that *P*_*m*_ « *P*_*f*_ and *P*_*S*_. The influence of *P*_*f*_ and *P*_*S*_ can be approximated using diffusion models (Karlsson and Artursson, 1991 and Artursson, 1991; Avdeef et al., 2005), or reduced by stirring (decreasing 1/*P*_*S*_; Cecchelli et al., 1999; Youdim et al., 2003; Summerfield et al., 2007) and using a transwell filter with larger pores (decreasing 1/*P*_*f*_; Siflinger-Birnboim et al., 1987; Adson et al., 1994). Alternatively, the sum of 1/*P*_*f*_ and 1/*P*_*S*_ can be measured in a control experiment with no cells on the transwell membrane.

Permeability coefficients obtained using the transwell assay are typically in the range from 10^−7^ to 10^−3^ cm s^−1^ (Pardridge et al., 1990; Deli et al., 2005; Summerfield et al., 2006, 2007), somewhat higher than values measured *in vivo*. Typical *P*_2D_ values for marketed CNS drugs vary between 10^−7^ and 10^−5^ cm s^−1^ (Summerfield et al., 2007). The measured permeability coefficient *P*_2D_ increases with lipophilicity reaching a plateau around log*P*_oct_ = 3. Increasing the lipophilicity above logP_oct_ = 3 results in a reduction of *P*_2D_ (Summerfield et al., 2007).

In general, *P*_2D_ increases approximately linearly with increasing lipophilicity. Deviations from this trend are generally due to violation of the assumption that transport is dominated by passive diffusion across the cell membrane. Apparent increases in *P*_2D_ can result from active transport and apparent decreases may be due to the influence of efflux pumps. At higher lipophilicities, solute trapping in the cell membrane and internal vesicles can lead to a lower apparent solute concentration in the output chamber and hence a lower apparent permeability.

### Resected vessel assay

As described above, the *in vitro* transwell assay is widely used to study passive transport across BMECs. However, the transwell assay has limited utility in studying active transport, since it is difficult to recapitulate the physiological polarization of pumps and transporters. A complementary method that is particularly useful in studying efflux pumps is the resected vessel assay (Schramm et al., 1995; Miller, 2003; Hartz et al., 2004; Bauer et al., 2005; Wang et al., 2010; Campos et al., 2012). In this assay, a resected brain capillary, typically from a rat or mouse brain, is transferred to a dish and immersed in buffer. A fluorescently-labeled solute of interest is then introduced into the media and uptake is measured by recording the fluorescence in the lumen of the vessel. The solute is usually a substrate for a particular transporter. For example, NBD-CSA is a fluorescent derivative of cyclosporine A that is a substrate for the P-gp pump (Didier et al., 1996), BODIPYFL prazosin is a fluorescently labeled substrate for BCRP (Robey et al., 2001), and Texas red is a fluorescent substrate for MRP2 (Bauer et al., 2008).

In the resected vessel assay, the solute is introduced into the bath, corresponding to the brain side of the vessel. The solute is transported across the abluminal membrane, diffuses across the cell, and then is transported across the luminal membrane. In general, the solute concentration in the lumen increases with time and reaches a steady state value after 30–60 min. Common efflux pumps such as P-gp and BCRP1 are expressed preferentially on the luminal membrane, therefore the concentration of the solute in the lumen of the vessel can be larger than in the bath. Since the direction of transport is from the brain parenchyma side to the vessel lumen, inhibition of efflux pumps is expected to decrease the solute concentration in the vessel.

**Figure 7:**
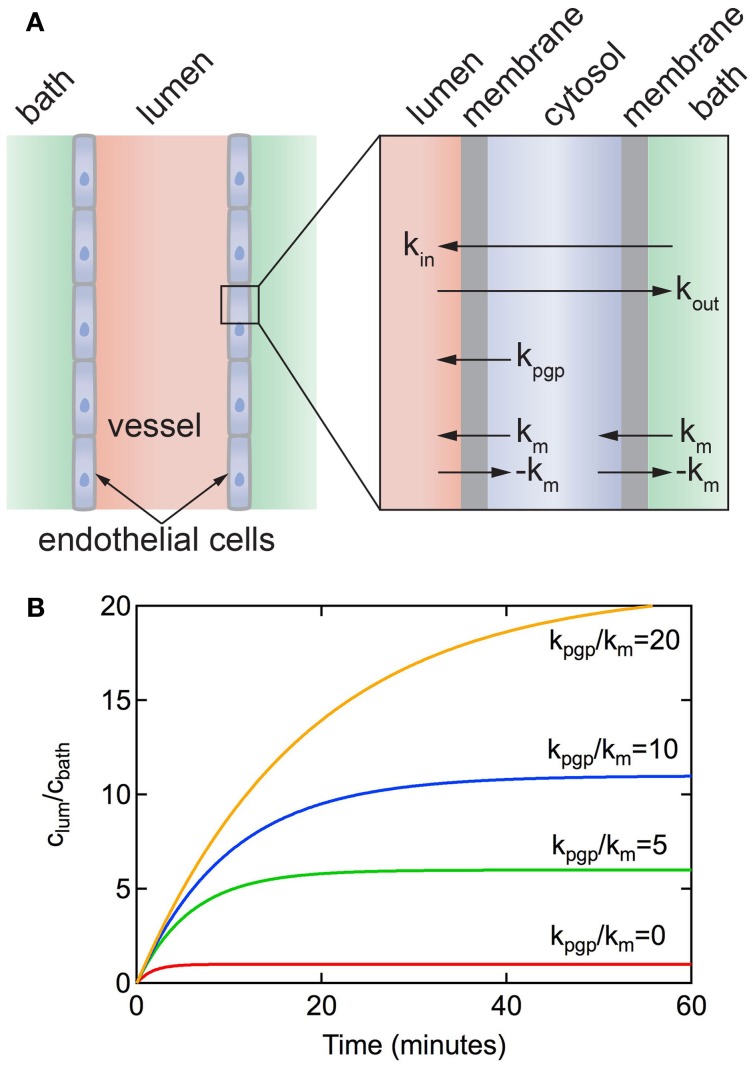
**(A)** Schematic illustration of a resected brain capillary defined by a layer of endothelial cells immersed in a bath. *k*_*m*_ is the rate constant for passive diffusion across a cell membrane. It is implicitly assumed that passive diffusion across the apical and luminal membranes is the same. *k*_pgp_ is the rate constant for active transport via efflux pumps from the cell to the lumen. It is assumed that active transport at the apical membrane is negligible. *k*_in_ and *k*_out_ represent the overall rate constants for transport from bath to lumen and lumen to bath, respectively. **(B)** Accumulation of solute in the lumen for a resected capillary with diameter *d* = 5 μm, *P*_3D_ = 3 × 10^−7^ cm s^−1^, *k*_pgp_/*k*_*m*_ = 0, 5, 10, 20.

Transport across the endothelium of a resected vessel can be analyzed using the model in Figure 7A (Ye and Searson, unpublished). Assuming that intracellular transport is fast in comparison to passive transport across the membrane that the forward and backward rate constants for passive diffusion are the same (*k*_*m*_ = *k*_−*m*_), and that the solute concentration inside the cell is approximately constant, we obtain (see Supplementary Information):

(9) $$c_{\text{lum}}\left(t\right)=c_{\text{bath}}\left(1+\frac{k_{\text{pgp}}}{k_{m}}\right)\left(1-\exp\left(-\frac{t}{\tau }\right)\right)$$

where the time constant τ is given by:

(10) $$\tau =\frac{r}{2k_{m}}\left(2+\frac{k_{\text{pgp}}}{k_{m}}\right)$$

Figure 7B shows plots of *c*_lum_(*t*)/*c*_bath_ vs. time for different values of *k*_pgp_/*k*_*m*_ for a vessel obtained from Equation 9. The concentration increases exponentially with time up to a steady-state value that is dependent on the ratio of *k*_pgp_/*k*_*m*_. Note that without the P-gp transporter (i.e., *k*_pgp_ = 0), *c*_lum_(∞)/*c*_bath_ = 1 and *k*_in_ = *k*_out_ = *k*_*m*_/2. As long as *k*_pgp_/*k*_*m*_ > 0 then *c*_lum_(∞)/*c*_bath_ > 1. The time to reach a steady state solute concentration in the lumen increases with increasing *k*_pgp_/*k*_*m*_. Results from experiments reported in the literature show a time to reach steady state of 30–60 min (Hartz et al., 2004; Bauer et al., 2007; Hawkins et al., 2010; Cannon et al., 2012), consistent with *k*_pgp_/*k*_*m*_ ≈ 5.

From examination of the model it can be seen that rate constant *k*_*m*_ = 2*P*_0_, where *P*_0_ is the permeability for passive diffusion across the cell. *P*_0_ can be equated to values obtained from *in vitro* transwell experiments or *in vivo* perfusion experiments where transport is dominated by passive diffusion. Typical values for *P*_0_ range from 10^−8^ to 10^−4^ cm s^−1^ (Summerfield et al., 2007).

### 3D transport

Various *in vivo* techniques, such as intravenous injection, *in situ* brain perfusion, microdialysis, and positron emission tomography (PET), have been employed to determine the kinetics of drug transport across the BBB (Takasato et al., 1984; Ungerstedt, 1991; Pike, 2009; Kuhnline Sloan et al., 2012). Brain perfusion in rats is the most widely used technique for obtaining *in vivo* permeability values for small molecules and drugs (Hammarlund-Udenaes et al., 2009). Brain perfusion allows injection of a solute into the brain vasculature at higher flow rates and solute concentrations than can be achieved through systemic circulation and hence allows a wider range of solute permeabilities to be measured at a fixed perfusate concentration (Takasato et al., 1984; Hammarlund-Udenaes et al., 2009). Direct injection of the solute into the brain minimizes metabolic loss and plasma protein binding (Takasato et al., 1984). In this technique, the main blood supply leading to an animal's brain, often the common carotid artery (either left or right), is cannulated and connected to a perfusion system. Immediately after the animal's heart is stopped, a molecule of interest dissolved in physiological perfusate solution is infused into its brain typically for 5–300 s. Subsequently, the brain is removed and the ipsilateral hemisphere is dissected, weighed, and the solute concentration determined by LC-MS, HPLC, GC, or scintillation counting if the solute is radiolabeled (Smith, 2003).

The rate constant for *in vivo* transport (*k*_in, 3D_) can be obtained from the measured solute concentration in the brain *Q*_br_ (g_s_ g_br_^−1^; see Figure 5):

(11) $$Q_{br}=k_{\text{in},\text{ 3D}}c_{p1}t$$

where *c*_pl_ is the solute concentration in plasma (g_s_ cm^−3^). In the derivation of Equation 11 it is assumed that: (1) the concentration of solute in plasma (*c*_pl_) is constant and (2) the flux of solute out of the brain is not significant over the short infusion period (i.e., k_out, 3D_c_br_ ≈ 0), which implies unidirectional transport (see Supplemental Information).

The rate constant, *k*_in, 3D_, is obtained from a single measurement of *Q*_br_/*c*_pl_ at a fixed infusion time *t*. The assumption of unidirectional transport can be confirmed by performing multiple perfusion experiments as a function of infusion time and determining the slope (*k*_in, 3D_) of a linear regression to a plot of *Q*_br_/*c*_pl_ vs. time (Pathak et al., 2011).

While *k*_in, 3D_ can be used to compare the *in vivo* transport kinetics of different solutes (Youdim et al., 2003), it cannot be compared directly to *in vitro* measurements *k*_in, 2D_. The rate constant *k*_in, 3D_ is related to the permeability *P*_3D_ through the Crone–Renkin equation (see Supplemental Information) (Renkin, 1959; Crone, 1963):

(12) $$k_{\text{in},\text{ 3D}}=F\left(1-\exp\left(\frac{P_{\text{3D}}S}{F}\right)\right)$$

where *F* is the normalized flow rate (cm^3^ s^−1^ g_br_^−1^) and *S* is the normalized luminal surface area of vessels (cm^2^ g_br_^−1^) in the brain. For the case where the flow rate *F* » *P*_3D_*S*, which is equivalent to the initial assumption that the plasma concentration of the solute *c*_pl_ is constant, the exponential term can be linearized and hence:

(13) $$k_{\text{in},\text{ 3D}}=P_{\text{3D}}S$$

Experimentally, as long as *F* ≥ 5*P*_3D_*S*, then the error in measurement of *P*_3D_ using Equation 13 is ≤ 10% (Smith and Takasato, 1986; Smith and Allen, 2003). S is taken to be 100–240 cm^2^ g_br_^−1^, as determined by morphometric analysis of rat brain tissue sections (Gross et al., 1986; Fenstermacher et al., 1988).

Typical values of *P*_3D_ for vascular tracers, nutrients, and drug molecules vary over 4 orders of magnitude from 10^−8^ to 10^−4^ cm s^−1^ (Takasato et al., 1984; Liu et al., 2004; Youdim et al., 2004; Summerfield et al., 2007). For small hydrophilic molecules, such as mannitol and sucrose, *P*_3D_ is typically in the range 10^−8^−10^−7^ cm s^−1^. In contrast, top selling antipsychotics and antidepressants such as venlafaxine, risperidone, buproprion, are generally small lipophilic molecules with *P*_3D_ values between 10^−5^ and 10^−4^ cm s^−1^ (Summerfield et al., 2007). Caffeine has an intermediate lipophilicity (log*P*_oct_ = −0.08) but relatively high permeability (*P*_3D_ = 4.2 × 10^−5^ cm s^−1^; Liu et al., 2004). Similarly, ethanol has an intermediate lipophilicity (log*P*_oct_ = −0.3) but a high permeability (*P*_3D_ = 1.1 × 10^−4^ cm s^−1^; Ohno et al., 1978; Takasato et al., 1984; Gratton et al., 1997).

**Figure 8:**
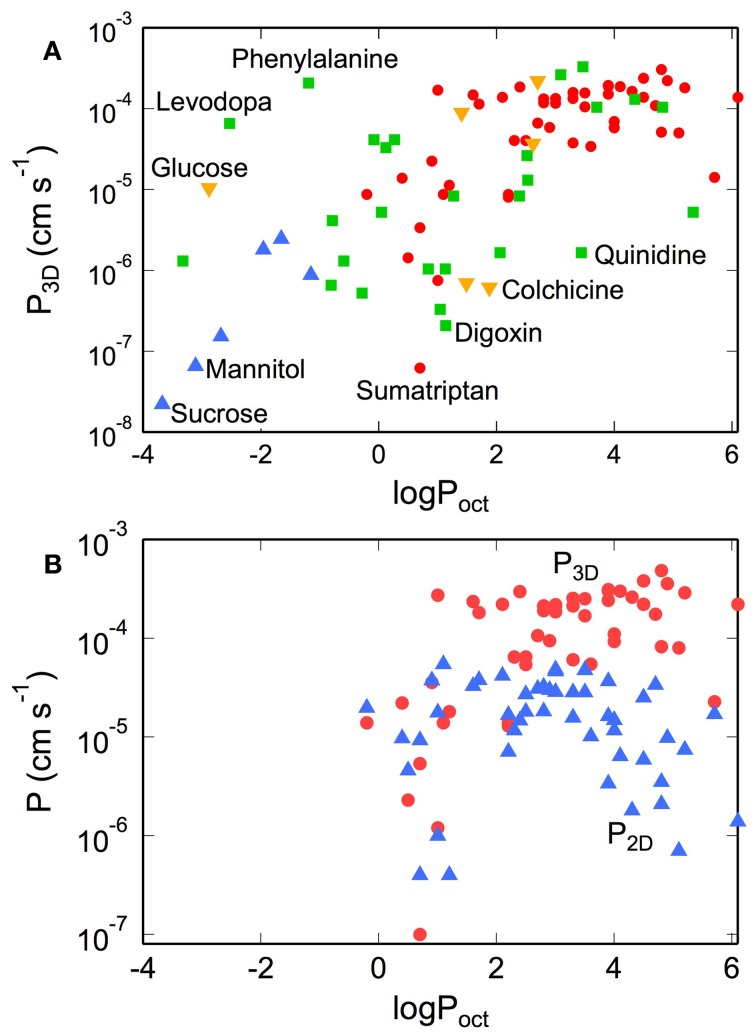
**(A)** Permeability of tracers, nutrients, and drugs obtained from *in situ* rat brain perfusion vs. lipophilicity. (•) Summerfield et al. (2007), (▲) Takasato et al. (1984), (▼) Youdim et al. (2004), (■) Liu et al. (2004) (**B)** Comparison of permeability of various CNS drugs obtained from transwell assays on monolayers of MDR1-MDCK (*P*_2D_) and *in situ* rat brain perfusions (*P*_3D_). *P*_3D_ values were obtained from *in situ* rat brain perfusion measurements reported in the literature. For data reported as the permeability surface area products (*P*_3D_*S*, cm^3^ s^−1^ g_br_^−1^) we take *S* = 150 cm^2^ g_br_^−1^. Values of *P*_3D_ where *S* ≠ 150 cm^2^ g_br_^−1^ were recalculated with *S* = 150 cm^2^ g_br_^−1^. Corresponding literature values for log*P*_oct_ were obtained from calculation Liu et al. (2004), Summerfield et al. (2007), and Youdim et al. (2004) or direct measurement of solute partitioning into octanol and water phases [Takasato et al. (1984)].

The *in vivo* 3D permeability for many small molecules increases linearly with lipophilicity up to log*P*_oct_ ≈ 3, implying that transport from the blood to the brain is dominated by passive transport across the cell membranes (see Figure 8A) (Ohno et al., 1978; Rapoport et al., 1979; Smith and Takasato, 1986; Lipinski et al., 2001; Liu et al., 2004; Summerfield et al., 2007; Zhao et al., 2009). Deviations from this behavior are indicative of other transport mechanisms (Lipinski et al., 2001). For example, D-glucose has a very low lipid solubility (log*P*_oct_ ≈ −3), but exhibits a high permeability coefficient (*P*_3D_ ≈ 10^−5^ cm s^−1^) since transport is facilitated by the GLUT-1 transporter. Conversely, colchicine has relatively high lipid solubility (log*P*_oct_ ≈ 2) but has a low permeability coefficient (*P*_3D_ ≈ 10^−6^ cm s^−1^), since it is a substrate of the P-gp efflux pump (Youdim et al., 2003; Liu et al., 2004). Morphine has a relatively low permeability (*P*_3D_ = 1.1 × 10^−6^ cm s^−1^) by drug standards, in part because it is a substrate for the P-gp pump (King et al., 2001), but highlights the fact that relevant doses can be achieved over reasonable time scales (Bouw et al., 2000; Tunblad et al., 2003; Hammarlund-Udenaes et al., 2008). Codeine (methyl morphine) has an -OH group on morphine substituted by a -O-CH_3_ group, resulting in an increase in log*P*_oct_ from 0.2 to 1.24, and increased permeability (Bostrom et al., 2008; Hammarlund-Udenaes et al., 2008). Dopamine has an intermediate lipophilicity (log*P*_oct_ = 0.84) but low permeability (*P*_3D_ = 1.1 × 10^−6^ cm s^−1^). However, L-dopa, a precursor that is metabolized to dopamine in the brain, has a very low lipophilicity (log*P*_oct_ = −2.53) but high permeability (*P*_3D_ = 6.6 × 10^−6^ cm s^−1^) since it is a substrate for the LAT-1 transporter (Gratton et al., 1997).

For log*P*_oct_ ≥ 3, both the apparent *in vitro* and *in vivo* permeabilities reach a maximum at about 10^−4^ cm s^−1^ (see Figure 8B) (Lipinski et al., 2001; Summerfield et al., 2007; Zhao et al., 2009). There are several factors that contribute to this apparent maximum. For solutes with high lipophilicity, transport becomes flow-limited. For *in situ* brain perfusion, the plasma concentration of the solute c_pl_ remains constant as long as *F* » *P*_3D_*S* (or *P*_3D_ « *F/S*). Since the maximum flow rate is typically around 0.2 cm^3^ s^−1^ g_br_^−1^, and assuming a luminal surface area of 100–200 cm^2^ g_br_^−1^, the plasma concentration *c*_pl_ is expected to remain constant for values of permeability up to *P*_3D_ ≈ 10^−4^ cm s^−1^. At this limit and above, most of the injected solute is absorbed into the brain, and as a result, the *in vivo* permeability does not increase with lipophilicity for log*P*_oct_ > 3. For *in vitro* measurements, at high rates of uptake, the apparent permeability can become limited by transport across the boundary layer or the porous membrane, as described above, resulting in an apparent maximum in permeability.

A noticeable difference between *P*_2D_ and *P*_3D_ occurs at log*P*_oct_ > 3. While *P*_3D_ maintains a plateau in this regime, *P*_2D_ decreases with increasing log*P*_oct_ for both MDCK and Caco-2 models (Wils et al., 1994; Sawada et al., 1999; Summerfield et al., 2007). This effect can be explained by solute binding and absorption in the cell membrane (Kubinyi, 1977). As a result, fewer molecules are able to efflux from the endothelium into the output chamber, thus contributing to a decrease in the apparent permeability (*P*_2D_). In the transwell assay, the amount of solute bound to the cell membrane, termed membrane retention or association, can be substantial (Sawada et al., 1999; Avdeef, 2001, 2003; Youdim et al., 2003; Fujikawa et al., 2007).

**Figure 9:**
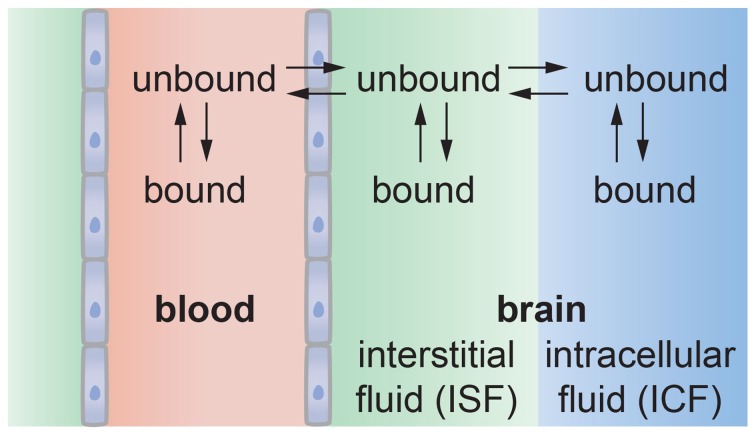
**Schematic illustration of solute transport from the vascular system into the brain**. The solute may bind with proteins or other components in blood that may reduce the amount that can enter the brain. Solute transported across the endothelium may be partitioned between the interstitial fluid and intracellular fluid in neurons and glial cells. Solute in the interstitial fluid may be bound to the ECM, reducing the amount available for uptake by cells.

For *in vivo* transport, solute binding or trapping can be more complicated (Figure 9). Solute that is transported across the brain endothelium can diffuse through the interstitial fluid in the ECM, as described previously, and ultimately be taken up by neurons or glial cells in the brain. However, solute in the interstitial fluid can also bind to the ECM and hence be unavailable therapeutically. Characterization of *in vivo* transport therefore requires knowledge about additional parameters. In one approach, the dynamics of solute transport *in vivo* can be captured by three parameters: *K*_*p, u*_, *P*_3D_, and *V*_*u*, brain_ (Hammarlund-Udenaes et al., 2008). *K*_*p, u*_ is the ratio of unbound drug in the brain and blood at steady state, and typically has values between 0.02–3. *K*_*p, u*_ = 1 for passive transport, *K*_*p, u*_ < 1 for active efflux, and *K*_*p, u*_ > 1 for active influx. *P*_3D_ describes the permeability of transport into the brain and can vary by four orders of magnitude (Figure 8). For passive transport, *P*_3D_ is expected to be related to the lipophilicity. The product *P*_3D_*S* (cm^3^ s^−1^ g_br_^−1^) corresponds to the net influx or clearance into the brain. *V*_*u*, brain_ (mL g_br_^−1^) is a measure of the distribution of the solute in the brain and is given by the ratio of the total amount of solute in the brain (mol g_br_^−1^) to the unbound concentration in the interstitial fluid (mol mL^−1^). If all of the solute is in the interstitial fluid and there is no solute in the brain cells, then *V*_*u*, brain_ = 0.2 mL g_br_^−1^, corresponding to the volume of interstitial fluid per gram in the rat brain. If the solute is uniformly distributed between the interstitial fluid and the intracellular fluid then *V*_*u*, brain_ = 0.8 mL g_br_^−1^, corresponding to the volume of water per gram in the rat brain. Values of *V*_*u*, brain_ > 0.8 mL g_br_^−1^, correspond to the case where the solute has affinity for brain tissue.

## The blood-brain barrier and disease

The barrier function of the BBB is critical for regulating transport to the brain, but also represents a significant roadblock in delivering drugs to the brain. Central nervous system diseases include mental disorders, migraine, epilepsy, neurodegenerative disease (e.g., Alzheimer's, Parkinson's, ALS, Huntington's), cerebrovascular disease (e.g., stroke), cancer, inflammatory disease (e.g., MS), trauma, and infections (e.g., meningitis; Hawkins and Davis, 2005; Hirtz et al., 2007; Neuwelt et al., 2008, 2011; Abbott et al., 2010; Daneman, 2012). Only very few CNS disorders such as depression, schizophrenia, chronic pain, and epilepsy are currently treatable with small molecule drug therapy. The BBB is the major roadblock in developing therapies for neurodegenerative diseases, cerebrovascular disease, inflammatory disease, infections, trauma, and brain cancer (de Boer and Gaillard, 2007; Pardridge, 2010).

Since the BBB is critical to maintain homeostasis in the brain, disruption can lead to changes in permeability, modulation of immune cell transport, and trafficking of pathogens into the brain (Hawkins and Davis, 2005; Engelhardt, 2008a,b; Abbott et al., 2010; Neuwelt et al., 2011). Disruption of the BBB is associated with many diseases of the central nervous system, including neurodegenerative diseases [e.g., Alzheimer's disease (Kalaria, 1999; Zlokovic, 2005; Desai et al., 2007; Zipser et al., 2007; Meyer et al., 2008; Hartz et al., 2010), ALS (Zhong et al., 2008), and Parkinson's disease (Kortekaas et al., 2005; Desai et al., 2007; Bartels et al., 2008)], cerebrovascular diseases [e.g., stroke (Belayev et al., 1996; Lippoldt et al., 2000; Lo et al., 2003; Del Zoppo, 2010; Moskowitz et al., 2010)], epilepsy and seizures (Seiffert et al., 2004; Oby and Janigro, 2006; Remy and Beck, 2006), brain infections [e.g., HIV encephalitis (Dallasta et al., 1999; Berger and Avison, 2004; Persidsky et al., 2006; Ivey et al., 2009) and meningitis (Uchiyama et al., 2009)], inflammatory diseases [e.g., MS (Kermode et al., 1990; Minagar and Alexander, 2003; Gold et al., 2006; Waubant, 2006; McQuaid et al., 2009)], brain tumors (Davies, 2002; Papadopoulos et al., 2004; Bronger et al., 2005), and neurotrauma (Stahel et al., 2000; Kim and Dustin, 2006; Shlosberg et al., 2010). There is also emerging evidence that mental or psychological stress may lead to local disruption of the BBB (Friedman et al., 1996). The association of BBB disruption with CNS diseases, suggests that BBB repair may prove to be an effective approach to maintain health and aid recovery from disease, infection, or injury (Abbott et al., 2010).

## Future perspectives

Drug delivery to the brain remains a major obstacle for treatment of CNS disorders. Advances in our understanding of the structure and function of the blood-brain barrier and development of innovative approaches for circumventing this barrier will be required to overcome the restricted access to brain circuits (Neuwelt et al., 2011). Moreover, there is an increasing appreciation that blood-brain barrier disruption contributes to the progression of central nervous system diseases. A key challenge is in understanding the dynamic response of barrier elements to focal disruptions and in developing strategies to accelerate repair.

While there is emerging insight into the formation of the blood-brain barrier during development (Daneman et al., 2010; Sohet and Daneman, 2013), very little is known about how aging affects barrier function. This is important, as the greatest risk factor for neurodegenerative disorders is aging. Insight into the morphology, turnover, dynamic behavior, and mechanical properties of endothelial cells during aging, as well as functional interactions with other cell types in the neurovascular unit will be required to define the role of BBB changes in both age-dependent cognitive decline and the progression of neurodegenerative diseases.

Central to advances in our scientific understanding of the BBB will be improved models for scientific and translational research. From an engineering perspective, the key features of the neurovascular unit are: (1) BMECs that function in a cylindrical geometry and experience shear stress resulting from blood flow, (2) functional interactions between BMECs, astrocytes, pericytes, other glial cells and neurons, (3) blood, which contains multiple cell types and soluble factors, and (4) 3D extracellular matrix and basement membrane. Determining how these features of the microvasculature interact will aid in the generation of BBB models compatible with high throughput screening methods that are likely to be crucial to the development of novel therapeutics.

### Conflict of interest statement

The authors declare that the research was conducted in the absence of any commercial or financial relationships that could be construed as a potential conflict of interest.
