# Supplementary material for: The blood-brain barrier: an engineering perspective
Source: Front Neuroeng. 2013 Aug 30;6:7. doi: 10.3389/fneng.2013.00007 (PMC3757302; doi:10.3389/fneng.2013.00007)
Supplement: Supplementary file 1 [file DataSheet1.PDF]

SUPPLEMENTAL INFORMATION
The Blood-Brain Barrier: An Engineering Perspective

Andrew D. Wong,[1,2] Mao Ye,[1,2] Amanda Levy,[1,2] Jeffrey D. Rothstein,[3] Dwight Bergles,[3] Peter C. Searson[1,2]

Appendix 1: TEER - resistance of blood plasma

Blood plasma makes up 55 - 60% of the total volume of blood. It is about 90% water by volume and contains various ionic and proteins:

Ion	Concentration (mM)
Sodium	135 - 145
Potassium	3.7 - 5.1
Chloride	95 - 105
Calcium	2.1 - 3.7
Carbonate	23 - 31
Phosphate	0.7 - 1.4

Protein	Concentration (mg mL^{-1})
Albumin	35 - 50
IgG	5 - 7
other	10 - 15

The conductivity of blood plasma can be estimated from the molar conductivity using the Debye-Huckel-Onsager equation:

$$\Lambda = \Lambda^\circ - (A\Lambda^\circ + B)\sqrt{c} \qquad \text{(A1.1)}$$

The molar conductivity at infinite dilution (Λ°) for NaCl is 161 cm^2 Ω^{-1} mol^{-1} at 37 ˚C. [1] Taking A = 1489 cm$^{5/2}$ Ω^{-1} mol$^{-1/2}$ and B = 6.83 cm$^{3/2}$ mol$^{-1/2}$ at 37 ˚C, the molar conductivity (Λ) for a 140 mM NaCl solution is 130 cm^2 Ω^{-1} mol^{-1}.

The resistivity can then be calculated from:

$$\rho_{\mathrm{pl}} = \frac{1}{\kappa} = \frac{1}{\Lambda c} \qquad \text{(A1.2)}$$

The resistivity for a 140 mM NaCl solution at 37 ˚C is 54.8 Ω cm, in good agreement with the experimentally measured resistance of blood plasma (54 - 62 Ω cm.[2]

The resistivity of whole blood depends on the fraction hematocrit:

$$\rho_{bl} = \frac{\rho_{pl}\left(1+\xi h\right)}{1-h} \tag{A1.3}$$

where ρ_{pl} is the resistivity of the blood plasma, h is the fraction hematocrit, and ξ is a form factor dependent on the shape of the red blood cells.[3]

Appendix 2: 2D transport

The net flux of a solute J (mole s^{-1}) across a barrier is the difference between the input and output fluxes:

$$J = \frac{dN}{dt} = k_{in}c_{in} - k_{out}c_{out} \tag{A2.1}$$

where N is the number of moles of solute in the output side, $k_{in,2D}$ and $k_{out,2D}$ are the first order rate constants (cm^3 s^{-1}), c_{in} and c_{out} (mole cm^{-3}) are the solute concentrations on the input and output sides, respectively.[4-6]

For diffusive transport it is seen that:[4-6]

$$J = \frac{dN}{dt} = -DA\frac{dc}{dx} \tag{A2.2}$$

where D is the diffusion coefficient (cm^2 s^{-1}) and A is the area (cm^2). The solute permeability is defined as the flux through unit area for unit concentration gradient:

$$P_{2D} = \frac{J}{A\Delta c} \tag{A2.3}$$

From Fick's first law the permeability coefficient can be related to the diffusion coefficient:

$$P_{2D} = \frac{D}{\Delta x} \tag{A2.4}$$

The flux can be written as:

$$J = \frac{dN}{dt} = -P_{2D}A\Delta c \tag{A2.5}$$

where $\Delta c = c_{out} - c_{in}$. Note that $N = c_{out}(t)V$, where V is the volume of the output side, and hence:

$$\frac{dc}{dt} = -\frac{P_{2D}A}{V}\left(c_{out}(t) - c_{in}\right)J \tag{A2.6}$$

Rearranging:

$$\frac{dc}{c_{in} - c_{out}(t)} = \frac{P_{2D}A}{V}dt \tag{A2.7}$$

Integrating:

$$c_{out}(t) = c_{in}\left(1 - \exp\left(-\frac{P_{2D}A}{V}t\right)\right) \quad \text{(A2.8)}$$

or

$$N(t) = Vc_{in}\left(1 - \exp\left(-\frac{P_{2D}A}{V}t\right)\right) \quad \text{(A2.9)}$$

For short times:

$$1 - \exp\left(-\frac{P_{2D}A}{V}t\right) = \frac{P_{2D}A}{V}t \quad \text{(A2.10)}$$

and hence:

$$c_{out}(t) = \frac{P_{2D}A}{V}c_{in}t \quad \text{(A2.11)}$$

or

$$N(t) = PAc_{in}t \quad \text{(A2.12)}$$

Appendix 3: resected vessel assay

We define N_{bath} as the number of moles of solute in the bath, N_{cell} as the number of moles of solute in the endothelium, and N_{lum} as the number of moles of solute in the lumen of the vessel. The concentration of solute in the bath is $c_{bath} = N_{bath}/V_{bath}$ and in the lumen is $c_{lum} = N_{lum}/V_{lum}$. As long as V_{bath} and $V_{lum} >> V_{cell}$, we can take show that:

$$\frac{dN_{lum}}{dt} = \frac{V_{lum}dc_{lum}}{dt} = k_{in}Ac_{bath} - k_{out}Ac_{lum} \quad \text{(A3.1)}$$

Rearranging:

$$\frac{dc_{lum}}{k_{in}Ac_{bath} - k_{out}Ac_{lum}} = \frac{1}{V_{lum}}dt \quad \text{(A3.2)}$$

Note that V_{lum}, k_{in}, k_{out}, and A are constants. We assume that $V_{bath} >> V_{lum}$, and hence c_{bath} is approximately constant. Integrating equation (A3.2) and recognizing that $c_{lum} = 0$ at $t = 0$, we obtain:

$$c_{lum}(t) = \frac{k_{in}}{k_{out}}\left(1 - \exp\left(-\frac{k_{out}A}{V_{lum}}t\right)\right)c_{bath} \quad \text{(A3.3)}$$

Substituting for k_{in} and k_{out}:

$$c_{lum}(t) = c_{bath}\left(1 + \frac{k_{pgp}}{k_m}\right)\left(1 - \exp\frac{t}{\frac{V_{lum}}{Ak_m}\left(2 + \frac{k_{pgp}}{k_m}\right)}\right) \quad \text{(A3.4)}$$

At long times, the concentration in the lumen reaches a steady state value given by:

$$c_{lum}(\infty) = \frac{k_{in}}{k_{out}} c_{bath} = \left(1 + \frac{k_{pgp}}{k_m}\right) c_{bath} \tag{A3.5}$$

Therefore, the time dependence of the concentration of solute in the lumen can be simplified as:

$$c_{lum}(t) = c_{lum}(\infty)\left(1 - \exp\frac{t}{\tau}\right) \tag{A3.6}$$

Appendix 4: in vivo transport

The net flux of a solute (J) between the brain and plasma is the difference between the flux into and out of the brain, Where the flux in and out from one compartment to another may be described as the product of a first order rate constant, $k_{in,3D}$ and $k_{out,3D,}$ and its respective concentration, c_{pl} and c_{br}.

$$J = \frac{dQ_{br}}{dt} = k_{in,3D} c_{pl} - k_{out,2D} c_{br} \tag{A4.1}$$

If we assume only a small amount of solute accumulates in the brain relative to the plasma ($Q_{br}/V_{br} << c_{pl}$), then we can ignore the back flux ($k_{out,3D} c_{br} \approx 0$).[7]

$$\frac{dQ_{br}}{dt} \approx k_{in,3D} c_{pl} \tag{A4.2}$$

Integrating equation A4.2, assuming that the concentration of solute in plasma (c_{pl}) is constant, we obtain

$$Q_{br} \approx k_{in,3D} c_{pl} t \tag{A4.3}$$

where t is the time of infusion, also known as the amount of time the brain is exposed to the drug. The net flux of solute into the brain can also be described by Fick's first law and related to the permeability coefficient (P_{3D}):

$$J = P_{3D} S\left(c_{pl} - c_{br}\right) \tag{A4.4}$$

Assuming the concentration of solute in the brain (c_{br}) is not significant over the duration of infusion ($c_{br} \approx 0$):

$$J \approx P_{3D} S c_{pl} \tag{A4.5}$$

where S is the normalized luminal surface area of vessels ($cm^2\ g_{br}^{-1}$) in the brain. Consider the mass balance of solute entering a length of brain microvessel (Figure A4.1). The total amount of solute that enters through the arterial end (x_a) is partitioned between diffusion out of a section of vessel with thickness Δx, and flow out of the venous end (x_v). Assuming that the back flux is negligible, the inflow of total solute ($g_s\ s^{-1}\ g_{br}^{-1}$) in plasma is equal to the sum of the flux into the brain parenchyma (equation A4) and the outflow of remaining solute in plasma ($g_s\ s^{-1}\ g_{br}^{-1}$). Inflow and outflow can be described as the product of concentration (c_{pl}) and flow rate (F) into

and out of the vessel. The flux into the brain is related to the permeability coefficient and the surface area and concentration (equation A4.5).

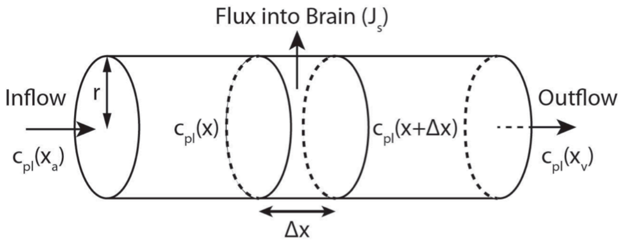

Figure A4.1. Illustration of solute mass balance.

From mass balance:

$$Fc_{pl}(x) = P_{3D}2\pi r\Delta x c_{pl}(x) + Fc_{pl}(x+dx) \tag{A4.6}$$

For small Δx (cm g_{br}^{-1}):

$$-F\frac{c_{pl}(x+dx) - c_{pl}(x)}{\Delta x} \approx -F\frac{c_{pl}(x)}{dx} = P_{3D}2\pi r c_{pl}(x) \tag{A4.7}$$

Rearranging and integrating along the vessel from the arterial end (x_a) to the venous end (x_v) we obtain:

$$c_{pl}(x_v) = c_{pl}(x_a)\exp\left(-\frac{P_{3D}S}{F}\right) \tag{A4.8}$$

where $\int_{x_a}^{x_v} 2\pi r\, dx = S$

The amount of solute that enters the brain (Q_{br}) over an infusion time of t with a given flow rate F is:

$$Q_{br} = \left(c_{pl}(x_a) - c_{pl}(x_v)\right)Ft \tag{A4.9}$$

Substituting equation A4.8 into equation A4.9 yields:

$$\frac{Q_{br}}{t} = c_{pl}(x_a)\left[1 - \exp\left(-\frac{P_{3D}S}{F}\right)\right]F \tag{A4.10}$$

From equation A4.3, we see that:

$$k_{in,3D} \approx \frac{Q_{br}c_{pl}}{t_0} \tag{A4.11}$$

Substituting equation A4.10 into A4.11 we obtain the Crone-Renkin Equation,[8,9] which relates $k_{in,3D}$ to P_{3D}.

$$k_{in,3D} = F\left[1 - \exp\left(-\frac{P_{3D}S}{F}\right)\right] \tag{A4.12}$$

If $P_{3D}S >> F$, then the exponential term can be linearized:

$$1 - \exp\left(-\frac{P_{3D}S}{F}\right) \approx \frac{P_{3D}S}{F} \tag{A4.13}$$

Combining equations A4.12 and A4.13, we obtain:

$$k_{in,3D} \approx P_{3D}S \tag{A4.14}$$

This equation is valid for short infusion periods over which back flux is negligible. Alternatively, equation A4.14 can derived using equation A4.3 to approximate the net flux of solute into the brain:

$$J = \frac{Q_{br}}{t} \approx k_{in,3D}c_{pl} \tag{A4.15}$$

Combining equations A4.15 and A4.4 and simplifying yields equation A4.14.

References

1. McCleskey, R. B. Electrical Conductivity of Electrolytes Found In Natural Waters from (5 to 90) degrees C. *J Chem Eng Data* **56**, 317-327 (2011).
2. Geddes, L. A.; Sadler, C. Specific Resistance of Blood at Body-Temperature. *Med Biol Eng* **11**, 336-339 (1973).
3. Sandberg, K.; Sjoqvist, B. A.; Olsson, T. Relation between Blood Resistivity and Hematocrit in Fresh Human-Fetal Blood. *Pediatr Res* **15**, 964-966 (1981).
4. Kedem, O.; Katchalsky, A. Thermodynamic analysis of the permeability of biological membranes to non-electrolytes. *Biochimica et biophysica acta* **27**, 229-246 (1958).
5. Siflinger-Birnboim, A.; Delvecchio, P. J.; Cooper, J. A.; Blumenstock, F. A.; Shepard, J. M.; Malik, A. B. Molecular-Sieving Characteristics of the Cultured Endothelial Monolayer. *J Cell Physiol* **132**, 111-117 (1987).
6. Dawson, D., Principles of membrane transport, in *Handbook of physiology, section 6: the gastrointestinal system*, ed. B. Rauner, (American Physiological Society: Bethesda, MD, 1991) 1-45.
7. Rapoport, S. I.; Ohno, K.; Pettigrew, K. D. Drug Entry into the Brain. *Brain Research* **172**, 354-359 (1979).
8. Crone, C. The Permeability of Capillaries in Various Organs as Determined by Use of the Indicator Diffusion Method. *Acta Physiol. Scand.* **58**, 292-305 (1963).
9. Renkin, E. M. Transport of Potassium-42 from Blood to Tissue in Isolated Mammalian Skeletal Muscles. *Am J Physiol* **197**, 1205-1210 (1959).
